# Population level usage of health services, and HIV testing and care, prior to decentralization of antiretroviral therapy in Agago District in rural Northern Uganda

**DOI:** 10.1186/s12913-015-1194-4

**Published:** 2015-11-28

**Authors:** G. Abongomera, S. Kiwuwa-Muyingo, P. Revill, L. Chiwaula, T. Mabugu, A. Phillips, E. Katabira, V. Musiime, C. Gilks, A. Chan, J. Hakim, R. Colebunders, C. Kityo, D. M. Gibb, J. Seeley, D. Ford, for the Lablite Project Team

**Affiliations:** Joint Clinical Research Centre, Kampala, Uganda; University of Antwerp, Antwerp, Belgium; Medical Research Council/Uganda Virus Research Institute, Entebbe, Uganda; University of York, York, UK; Dignitas International, Zomba, Malawi; University of Zimbabwe Clinical Research Centre, Harare, Zimbabwe; University College London, London, UK; Infectious Diseases Institute, Makerere University, Kampala, Uganda; Makerere University College of Health Sciences, Kampala, Uganda; Imperial College London, London, UK; School of Population Health, University of Queensland, Brisbane, Australia; Division of Infectious Diseases, Department of Medicine, University of Toronto, Toronto, Canada; Medical Research Council Clinical Trials Unit at UCL, London, UK; London School of Hygiene and Tropical Medicine, London, UK

**Keywords:** Sub-Saharan Africa, Uganda, Health service usage, HIV services, Antiretroviral therapy rollout, Population survey

## Abstract

**Background:**

Decentralization of ART services scaled up significantly with the country wide roll out of option B plus in Uganda. Little work has been undertaken to examine population level access to HIV care particularly in hard to reach areas in rural Africa. Most work on ART scale up has been done at health facility level which omits people not accessing healthcare in the community. This study describes health service usage, particularly HIV testing and care in 2/6 parishes of Lapono sub-county of northern Uganda, prior to introduction of ART services in Lira Kato Health Centre (a local lower-level health centre III), as part of ART decentralization.

**Methods:**

Household and individual questionnaires were administered to household members (aged 15–59 years). Logit random effects models were used to test for differences in proportions (allowing for clustering within villages).

**Results:**

2124 adults from 1351 households were interviewed (755 [36 %] males, 1369 [64 %] females). 2051 (97 %) participants reported seeking care locally for fever, most on foot and over half at Lira Kato Health Centre. 574 (76 %) men and 1156 (84 %) women reported ever-testing for HIV (*P* < 0.001 for difference); 34/574 (6 %) men and 102/1156 (9 %) women reported testing positive (*P* = 0.04). 818/850 (96 %) women who had given birth in the last 5 years had attended antenatal care in their last pregnancy: 7 women were already diagnosed with HIV (3 on ART) and 790 (97 %) reported being tested for HIV (34 tested newly positive). 124/136 (91 %) HIV-positive adults were in HIV-care, 123/136 (90 %) were taking cotrimoxazole and 74/136 (54 %) were on ART. Of adults in HIV-care, most were seen at Kalongo hospital (*n* = 87), Patongo Health Centre (*n* = 7) or Lira Kato Health Centre (*n* = 23; no ART services). 58/87, 5/7 and 20/23 individuals walked to Kalongo hospital (56 km round-trip, District Health Office information), Patongo Health Centre (76 km round-trip, District Health Office information) and Lira Kato Health Centre (local) respectively. 8 HIV-infected children were reported; only 2 were diagnosed aged <24 months: 7/8 were in HIV-care including 3 on ART.

**Conclusions:**

Higher proportions of women compared to men reported ever-testing for HIV and testing HIV-positive, similar to other surveys. HIV-infected men and women travelled considerable distances for ART services. Children appeared to be under-accessing testing and referral for treatment. Decentralization of ART services to a local health facility would decrease travel time and transport costs, making care and treatment more easily accessible.

**Electronic supplementary material:**

The online version of this article (doi:10.1186/s12913-015-1194-4) contains supplementary material, which is available to authorized users.

## Background

Seventy percent of HIV-infected people live in Sub Saharan Africa [1] and in Uganda, the estimated HIV prevalence was 7.3 % in 2013 [2]. By the end of 2013, nearly 600,000 people were receiving antiretroviral therapy (ART), corresponding to ART coverage of approximately 69 % [3]. The recent change in WHO guidelines raising the CD4 threshold for ART initiation from ≤350 to ≤500 cells/mm^3 ^increases the estimated number of individuals still eligible for ART by a further 50 % [4].

In Uganda, health facilities are categorized by the area served and services provided: a) Health Centre II (HC II) serves a parish with population ~5,000 and provides outpatient, antenatal, immunization and outreach services; b) Health Centre III (HC III) serves a sub-county and additionally provides inpatient care and environmental health; c) Health Centre IV (HC IV) serves a sub-district and additionally provides surgery, supervision of the lower HCs, data collection and health service planning [5]. Decentralization of ART services to HC IIIs nearer to where people live scaled up significantly with the countrywide roll out of Option B plus (initiation of all pregnant and breastfeeding women on ART for life) which commenced in September 2012 [6]. Prior to this, ART services were predominantly available in hospitals and HC IVs, and ART provision in most HC IIIs was limited to individual antiretroviral drugs for prevention of mother-to-child transmission (pMTCT), with no provision of fixed-dose combination ART for treatment.

Studies conducted in Malawi and South Africa on scale-up of ART services in rural areas report reductions in adult mortality subsequent to ART roll-out [7–9]. However, such scale-up is often inequitable between rural and urban settings, due to severe shortages of health workers in rural settings, even where task-shifting to lower cadres for delivery of ART is undertaken, as recommended by WHO [10–12]. Little work has been undertaken to examine population-level access to HIV care in hard-to-reach rural areas in Africa, or to describe the population-level effects of decentralizing ART. Most work has been based on sampling at health facilities, which omits those in the community not accessing healthcare [13, 14]. Information on population-level access and uptake of HIV testing, care and treatment is important for planning services and for understanding barriers to achieving ART coverage targets.

Lablite is an implementation project working in partnership with Ministries of Health to investigate and support strategies to roll-out HIV treatment safely and cost-effectively to primary care health centres in rural settings in Uganda, Malawi and Zimbabwe [15]. In Uganda, Lablite is being implemented in two districts including the Agago District in Northern Uganda and Kalungu District in central Uganda. People of Northern Uganda were victims of a rebel insurgency led by Joseph Kony of the Lord’s Resistance Army (LRA) for more than 20 years prior to the successful peace process initiated in 2007. It was characterized by child abductions, frequent rapes, attacks on civilian camps and killing [16]. According to the Uganda demographic and health survey (UDHS) 2011, the poverty index is higher in the northern region than elsewhere in the country and sanitation, transport, infrastructure and healthcare services are often lacking [17].

Agago District in northern Uganda has an estimated total population of 299,700 [18]. Lapono Sub County (study area) is one of the 13 sub-counties with an estimated population of 22,785 [19]. Lira Kato HC III is the highest level health facility in this sub county and provides outpatient services, inpatient services for children, HIV/AIDS counselling and testing (HCT), antenatal care (ANC), maternity and deliveries, pMTCT, immunizations and limited laboratory services. Prior to accreditation for ART provision in November 2013, people from Lapono Sub County who needed ART mainly attended Patongo HC III and Kalongo Hospital located 38 km and 28 km from Lapono sub county respectively (distances provided by Agago District Health Office).

The objective of this study was to describe health service usage in the villages surrounding a health centre III, particularly HIV testing and care, prior to planned ART decentralization.

## Methods

**Fig. 1:**
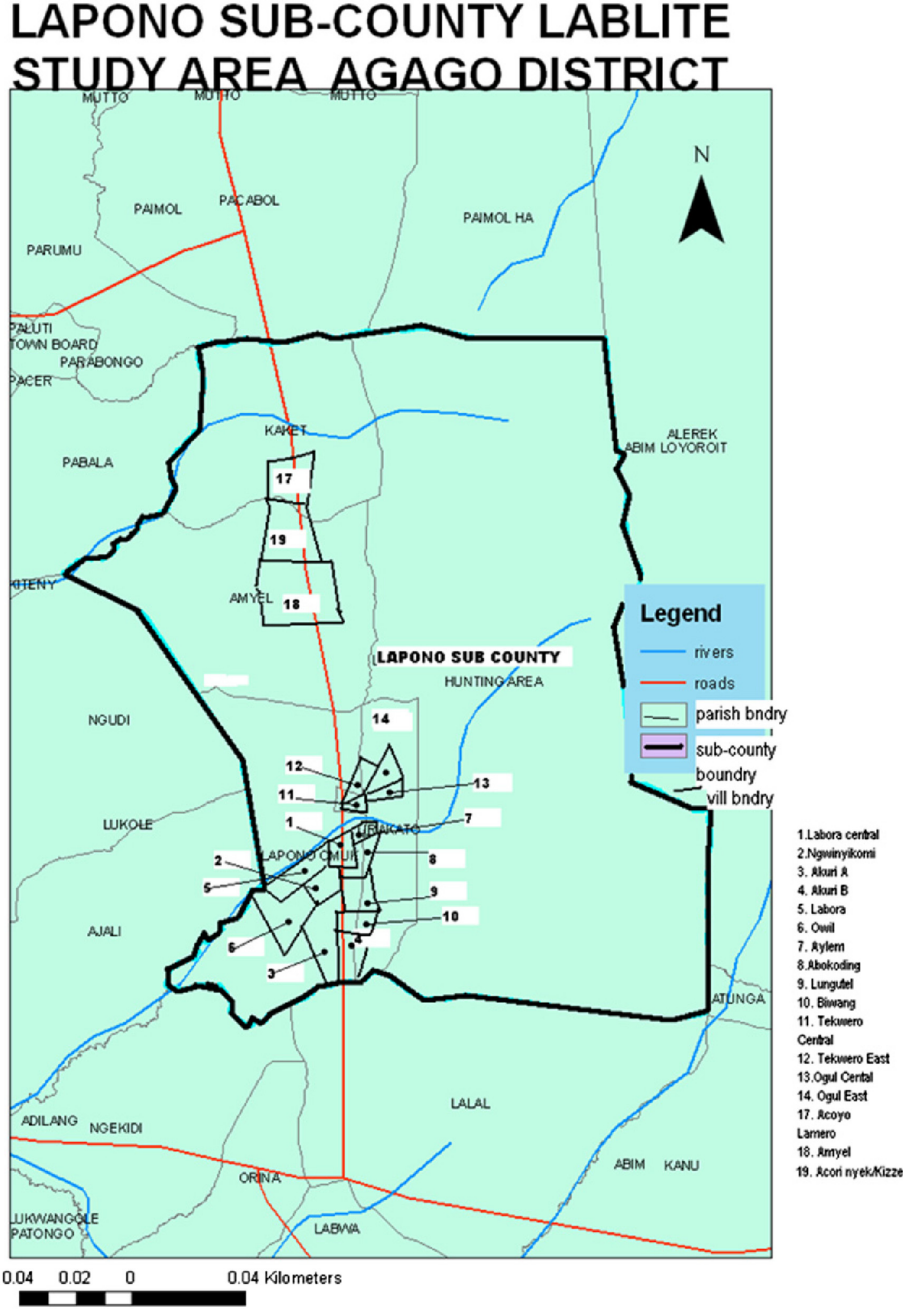
Map of Lapono sub-county, dots indicate villages included in the survey (village 1 = pilot)

We conducted a cross-sectional population based survey in 2 of the 6 parishes including ~20 % of villages in Lapono sub-county (Fig. 1) between February and April 2013 prior to decentralization of ART at Lira Kato HC III. The survey was planned to include at least 100 HIV-infected adults; based on an HIV-prevalence of 8 and 40 % of HIV-infected adults knowing and reporting their status correctly [2], we anticipated interviewing ~3,000 adults. Data were monitored regularly and the survey was stopped early after 2 parishes had been completed and >100 HIV-infected adults had been identified.

All villages in the two parishes closest to Lira Kato HC III were included. One village was used for the pilot (excluded from main survey) and seventeen villages were included in the main survey. Every household in each village was mapped and approached during the survey. Participant engagement to encourage participation was through community meetings held in collaboration with local leaders. Village mapping was done using Geographical Positioning System (GPS) technology for fourteen villages and manual mapping for three villages, with additional guidance from a village member to locate dwellings and demarcate village boundaries within the study area.

### Data collection

The survey team included four members trained in data collection and fluent in Luo (local language), led by two field supervisors. Each household was visited by an interviewer accompanied by a local village member. If household members were not at home, the interviewer returned later; up to 2 visits were made to each household. The protocol included collection of individual demographic and health-related information from up to three adults aged 15–59 years in each household and socioeconomic data and information on children from the household head (or another adult) (Additional files 1 and 2). Interviews were conducted in 1459 households (~77 % of households mapped); the household questionnaire was completed for 1401. 2124 adults from 1351 households were interviewed (108 households had no age-eligible adult at home). Numbers interviewed per household were: 1 adult in 753 households; 2 adults in 454 households, 3 adults in 116 households; 4 or 5 adults in 28 households (more than permitted in the protocol). All data are reported.

### Statistical analysis

Analyses were conducted using Stata 12.1. Most analyses were descriptive. We tested for differences in knowledge about HIV and HIV prevention practice, HIV testing and reported HIV-infection between men and women because previous population surveys have shown differences by sex [2] and because women access HIV-testing through antenatal care (ANC). Logit random effects models were used to test for differences in proportions including a random effect for village.

### Ethics

The study protocol was approved by the Joint Clinical Research Centre/Research Ethical Committee (JCRC/REC) and from the Uganda National Council for Science and Technology (UNCST) and Office of the President of the Republic of Uganda. Written informed consent was obtained from all the study participants.

## Results

### Characteristics of study participants

Table 1 outlines study household socio-economic indicators compared with households sampled in the last Uganda Demographic and Health survey (UDHS)in 2011 [17]. In 742/1401 households the head of household was interviewed; 456/742 (62 %) were men. 1370 (98 %) households used a borehole as the main water source and 537 (38 %) households had a toilet; most of these were pit latrines (97 %). 1339 (96 %) used paraffin lanterns or candles as the predominant form of lighting. 844 (61 %) households owned a mosquito net, 349 (25 %) a radio, 4 (<1 %) a television and 395 (28 %) had a mobile phone. For transport, 497 (36 %) owned bicycles, 42 (3 %) motorcycles and 3 (<1 %) owned cars/trucks.

**Table 1:** Percent distribution of household characteristics in the UDHS 2011 and the current survey

Characteristic	UDHS 2011		Survey
	Urban	Rural	
	*n* = 2551	*n* = 6482	*n* = 1401^a^
Household Headship^b^			
Male	69.0	70.8	456 (61.5)
Female	31.0	29.2	286 (38.5)
Main water source^c^			
River	NA	NA	2 (0.1)
Well/Spring	12.5	28.4	10 (0.7)
Borehole	11.8	43.9	1370 (97.9)
Rain catchment	0.5	1.4	1 (0.1)
Water tap in house/plot	27.9	1.5	0 (0.0)
Trench	NA	NA	8 (0.6)
Dam	NA	NA	9 (0.6)
Stand pipe/public tap	38.9	8.2	NA
Bottle water	4.6	0.4	NA
Tanker truck/vendor	2.2	0.9	NA
Surface water	1.0	14.6	NA
Other	0.6	0.8	NA
Toilet			
Pit latrine	67.4	83.4	501 (35.8)
VIP latrine	18.6	4.2	12 (0.9)
Flush toilet	11.3	0.3	0 (0.0)
Other toilet	0.8	0.6	4 (0.3)
Toilet type not specified	0.0	0.0	20 (1.4)
No facility	1.8	11.5	864 (61.7)
Predominant Lighting^d^			
Electricity	55.9	3.9	11 (0.8)
Paraffin lantern/Candle	35.4	86.7	1339 (96.3)
Wax candle	5.8	2.0	5 (0.4)
Others	3.0	7.5	36 (2.6)
Household Possessions			
Mosquito Net	80.9	72.4	844 (60.8)
Radio	71.8	64.6	349 (25.0)
Television	45.0	4.9	4 (0.3)
Mobile phone	86.8	53.1	395 (28.3)
Means of transport			
Bicycle	19.5	41.1	497 (35.6)
Motorcycle	11.4	7.1	42 (3.0)
Car/truck	10.1	1.6	3 (0.2)

^a^For the PBS numbers and percentages of households are provided. Where numbers do not sum to total, this is due to missing data. Percentages are of non-missing data

^**b**^Not all participants interviewed for the PBS household questionnaire were heads of household and this information is only available if the head of household was interviewed

^c^The choices for water source did not completely overlap between the 2 surveys. NA indicates the choice was not available and zero indicates it was available but not selected

^d^Taken from Uganda AIDS Indicator Survey, 2011 [2] as not available in the UDHS Survey [17]

In total 2124 adults were interviewed including 755 (36 %) males and 1369 (64 %) females (Table 2). Median age of participants was 29.1 years and 1493 (70 %) were married. 1621 (77 %) had not completed primary school. Most individuals had more than one source of livelihood (median 2 sources), subsistence farming being the most common (2046 (97 %)), followed by livestock farming and growing crops to sell. 724 (53 %) of women brewed alcohol as a source of livelihood.

**Table 2:** Socio-demographic characteristics of the study participants

Characteristic	Men	Women	Total
	*n* = 755	*n* = 1369	*n* = 2124
Age			
≤ 19	208 (27.6)	298 (21.8)	506 (23.9)
20–29	195 (25.9)	416 (30.4)	611 (28.8)
30–39	194 (25.8)	384 (28.1)	578 (27.3)
40–49	108 (14.3)	169 (12.4)	277 (13.0)
50+	48 (6.4)	100 (7.3)	148 (7.0)
Highest level of Education			
None	44 (5.8)	520 (38.2)	564 (26.6)
Pre-primary/Some primary	387 (51.3)	670 (49.2)	1057 (49.9)
Completed primary	104 (13.8)	93 (6.8)	197 (9.3)
Some secondary	142 (18.8)	63 (4.6)	205 (9.7)
Completed secondary	25 (3.3)	10 (0.7)	35 (1.7)
Higher Education/Vocational	53 (7.0)	7 (0.5)	60 (2.8)
Source of Livelihood^1^			
Subsistence crop grower	710 (94.0)	1336 (97.6)	2046 (96.3)
Cash crop grower	322 (42.6)	573 (41.2)	895 (42.1)
Livestock farmer	309 (40.9)	476 (34.8)	785 (37.0)
Brick Maker	97 (12.8)	4 (0.3)	101 (4.8)
Alcohol brewing	0 (0.0)	724 (52.9)	724 (34.1)
Petty/retail business	68 (9.0)	192 (14.0)	260 (12.2)
Others	191 (25.3)	127 (9.3)	310 (14.6)
Current Partnership			
Married	493 (65.3)	1000 (73.0)	1493 (70.3)
Living with partner as if married	10 (1.3)	18 (1.3)	28 (1.3)
Never married	232 (30.7)	179 (13.1)	411 (19.4)
Widow/widower	6 (0.8)	109 (8.0)	115 (5.4)
Separated/Divorced	14 (1.9)	63 (4.6)	77 (3.6)

Data are n (%). Where numbers do not sum to total this is due to missing data. Percentages are of non-missing data

^1^Individuals were able to report >1 source of livelihood

### Use of health services for general healthcare

Most participants stated that for a fever they normally go to local health facilities, including Lira Kato HC III (1073 (51 %) and HC IIs (978 (46 %); only 44 (2 %) go to Kalongo Hospital (Table 3). The majority (97 %) individuals going to local health centres (Lira Kato HC III and HC IIs) travel on foot, and spend median (IQR) 4 (3–6) hours for the round trip, including waiting and receiving medical attention (Table 4). Approximately half of individuals visiting Kalongo Hospital also walk; reported time for the round trip when walking was median 10 (9–12) hours. Reported distance to Kalongo hospital was less than the actual 28 km, with reported time taken also lower than feasible; this suggests people may have stayed somewhere on the way or considerable under-estimation. Of 1101 participants who had had a serious illness in the last 12 months, 40 % went to Lira Kato HC III, 29 % went to a local HC II and 24 % went to Kalongo Hospital (Table 3).

**Table 3:** Usage of health facilities by study participants for non-HIV services

Healthcare facility	Facility normally visited for fever	Facility visited for last serious sickness
	*n* = 2122^a^	*n* = 1101^b^
Kalongo hospital	44 (2.1)	265 (24.1)
Patongo HC III	2 (0.1)	0 (0)
Lira Kato HC III	1073 (50.6)	441 (40.1)
Health centre II in sub-county	978 (46.1)	321 (29.2)
Other hospital/HC	13 (0.6)	31 (2.8)
Local private clinic	12 (0.6)	31 (2.8)
Traditional healers/herbalist	0 (0.0)	12 (1.1)

Data are n (%) of participants

^a^Missing data for 2 participants

^b^23 participants reported a serious sickness requiring medical care in the previous 12 months but facility missing

**Table 4:** Reported time taken for roundtrip on foot to facility for a fever including consultation time

Healthcare facility	Proportion travelling on foot^a^	Time taken for roundtrip on foot to facility in hours	Reported distance in kilometres (one way) by those who walked
		Median (IQR)	Median (IQR)
Kalongo hospital	21/43 (49 %)	10 (9–12)	12 (9–15)
Patongo HC III	1/2 (50 %)	10.5	6
Lira Kato HC III	1043/1071 (97 %)	5 (3–6)	1 (0.5–2)
Health centre II in sub-county	962/977 (98 %)	4 (3–5)	1 (0.8–2)
Other hospital/HC	11/12 (92 %)	4 (3–6)	1 (0.5–3)
Local private clinic	10/12 (83 %)	1 (0.5–1.5)	1 (0.5–2)

^a^Where denominators are less than total in Table 3 this is due to missing data. Percentages are of non-missing data

### Knowledge about HIV and HIV prevention practice

739 (98 %) of men and 1361 (99 %) of women had heard of HIV/AIDS (Table 5). Most rejected common misconceptions, although 173 (23 %) men and 435 (32 %) women thought HIV could be transmitted by mosquito or other insect bites. Most participants reported transmission of HIV was possible during child birth and breastfeeding; however 425 (56 %) men and 825 (60 %) women thought HIV transmission was not possible during pregnancy or were unsure. Of those who knew that mother to child transmission (MTCT) of HIV could occur, 497 (71 %) men and 994 (76 %) women knew drugs were available for mothers to take to reduce the risk. 723 (98 %) men and 1347 (99 %) women (who had heard of HIV/AIDS) reported practising HIV prevention methods, with the majority reporting being faithful to their partners. 368 (50 %) men reported condom use; this compares with 262 (19 %) women reporting condom use by their partner(s) (*P* < 0.001 for difference).

**Table 5:** Knowledge about HIV and HIV prevention practice

HIV Knowledge and Prevention Practices	Men			Women			Test for difference by sex^c^
	*n* = 755			*n* = 1369			
	Yes	No	Don’t know/not sure	Yes	No	Don’t know/not sure	
Have you heard of HIV or AIDS?	739 (97.9)	16 (2.1)		1361 (99.4)	8 (0.6)		0.003
Ways of transmitting HIV/AIDS^a^							
Can be transmitted by sex with someone who looks healthy	628 (83.3)	63 (8.4)	63 (8.4)	1084 (79.2)	122 (8.9)	162 (11.8)	0.03
Can’t be transmitted by mosquito or other insect bites	480 (63.8)	173 (23.0)	100 (13.3)	638 (46.6)	435 (31.8)	295 (21.6)	<0.001
Can’t be transmitted by bewitchment/curses or supernatural means	652 (86.6)	34 (4.5)	67 (8.9)	1159 (84.9)	56 (4.1)	151 (11.1)	0.28
Can’t be transmitted by sharing food with HIV infected person	656 (87.1)	36 (4.8)	61 (8.1)	1141 (83.4)	82 (6.0)	145 (10.6)	0.03
Can be transmitted by having sex without condom	726 (96.6)	4 (0.5)	21 (2.8)	1338 (98.0)	11 (0.8)	17 (1.2)	0.08
Knowledge about PMTCT^a^							
HIV can be transmitted to child during pregnancy	330 (43.7)	298 (39.5)	127 (16.8)	544 (39.7)	537 (39.2)	288 (21.0)	0.08
HIV can be transmitted to child during delivery	670 (88.7)	22 (2.9)	63 (8.3)	1265 (92.4)	34 (2.5)	70 (5.1)	0.005
HIV can be transmitted to child during breastfeeding	653 (86.5)	23 (3.1)	79 (10.5)	1248 (91.2)	30 (2.2)	91 (6.7)	0.001
Do you practise any HIV prevention methods?^b^	723 (97.8)	16 (2.1)		1347 (99.0)	14 (1.0)		0.04
HIV prevention practices used^b^							
Abstinence	260 (35.2)	477 (64.6)	1 (0.1)	478 (35.2)	880 (64.7)	2 (0.2)	0.99
Faithfulness	510 (69.0)	226 (30.6)	3 (0.4)	1044 (76.7)	316 (23.2)	1 (0.1)	<0.001
Condom use	368 (49.8)	371 (50.2)	0 (0.0)	262 (19.3)	1097 (80.7)	1 (0.1)	<0.001
Circumcision	21 (2.9)	685 (92.8)	32 (4.3)	5 (0.4)	1073 (78.8)	283 (20.8)	<0.001
Others	123 (18.5)	451 (67.9)	90 (13.6)	180 (14.3)	828 (66.8)	231 (18.6)	0.02

Data are n (%) of participants

^a^For all questions covering knowledge of modes of HIV transmission, a “yes” indicates correct knowledge. Participants who had not heard of HIV/AIDS (*n* = 24) were included under “don’t know/not sure”

^b^Participants who had not heard of HIV/AIDS (*n* = 24) were not asked about HIV prevention practice

^c^Test for difference compares yes vs. no/don’t know/not sure by logit random effects model

### History of HIV testing and reported HIV prevalence

574 (76 %) men and 1156 (84 %) women reported ever testing for HIV (*P* < 0.001 for difference). Testing rates were high and similar in men and women who had ever been married (478/523 (91 %) and 1094/1190 (92 %) respectively; *P* = 0.71). Rates were lower in both men and women who had never been married (96/232 (41 %) and 62/179 (35 %) respectively; *P* = 0.16 for difference by sex; *P* = 0.001 for difference by marital status). 406 (54%) men and 671 (49%) women reported testing in the last year (*P* =0.04 ).Of those who had had an HIV test 34/574 (6 %) men and 102/1156 (9 %) women reported testing positive (*P* = 0.03).

### HIV testing and PMTCT during pregnancy

Of 850 women who had given birth in the 5 years prior to survey, 818 (96 %) had attended antenatal care (ANC) at least once in the pregnancy leading to their most recent birth; 439 (52 %) women attended at least 4 times. The most common reason for not attending ANC was the husband refusing to accompany them to clinic (18/32). Median (IQR) for month of first ANC attendance was 4 (3–5) months. Of those who attended ANC, 7 women were already diagnosed with HIV (including 3 on ART) and 790 of the remaining 811 (97 %) reported being tested for HIV; 14/21 women not tested had not been offered a test. 34/790 (4 %) of women tested newly positive; however of these only 22/34 (65 %) reported receiving drugs for themselves and 23/34 (68 %) for their babies for PMTCT.

### HIV care and treatment

**Fig. 2:**
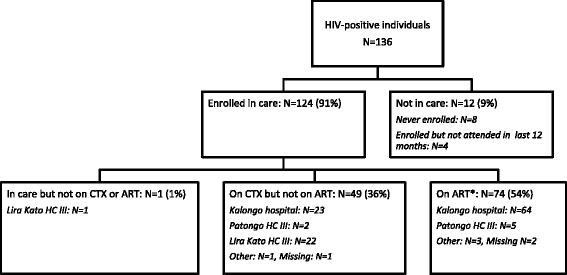
Care and treatment of self-reported HIV-infected adults identified in the survey. *3 patients at Kalongo hospital were on ART but not on CTX; remaining individuals were on CTX and ART. All percentages are of the total 136 HIV-positive individuals

Of 136 HIV-positive adults, 124/136 (91 %) were in HIV-care, 120/136 (88 %) were taking cotrimoxazole (CTX) and 74/136 (54 %) were on ART (Fig. 2). The12 individuals (2 men and 10 women) not in HIV care reported the following reasons: transport costs (3), lack of drugs (1), stigma (1), never referred (2), service costs (1) and other personal circumstances (4). 24 (32 %) individuals currently on ART had initiated ART within the last 12 months and 50 (68 %) more than 12 months previously. Seven individuals reported using ART in the past but were no longer taking ART. Of adults in HIV-care, 87 (70 %) were seen at Kalongo Hospital, 23 (17 %) went to Lira Kato HC III (no ART services), 7 (5 %) went to Patongo HC III, and 7 (6 %) went elsewhere or facility information was missing. 58/87, 20/23, 5/7 and 3/7 individuals travelled on foot to Kalongo hospital (56 km round-trip), Lira Kato HC (local), Patongo HC (76 km round-trip) and elsewhere respectively. Most of those not travelling on foot used a bicycle.

Most patients collected drugs 3 monthly: 65 (56 %) of those on CTX and 52 (69 %) on ART, with the remainder collecting drugs more frequently. There were differences by care facility: 17 (74 %) patients collected CTX monthly from Lira Kato compared with 7 (8 %) patients from Kalongo Hospital (*p* < 0.001). 14 (12 %) individuals on CTX and 6 (8 %) on ART reported travelling to clinic in the last year and being unable to collect drugs (10/14 and 4/6 respectively due to stock-outs at Lira Kato (5), Patongo (3), Kalongo (4), other (2), respectively). 15 (20 %) individuals on ART had sent someone else to collect their ART drugs once in the last 12 months and 13 (18 %) at least twice. 90 (73 %) of individuals in care had their blood taken in the last year for CD4 testing, including 24 (48 %) individuals not on ART.

### HIV-testing and treatment in children

Numbers of children per household ranged from 0 to 14, with median (IQR) of 3 (2–4) per household. In total, interviewees in 26 households reported that at least one child in the household had tested positive for HIV. In 2 households a child had been tested approximately 10 months previously (one at Kalongo Hospital and one at an HTC outreach) but results had not been received. In a further 30 households the interviewee reported that there was at least one child who they were worried might be HIV-infected but had not been tested. Reasons for not testing included: not wanting to know (*n* = 3); fear (*n* = 3); counselling offices too far (*n* = 3); not interested (*n* = 2); does not know where to take them (*n* = 9); no reason given (*n* = 10).

Information on care was provided for only 8 HIV-infected children in 7 households, of whom only 2/8 children were diagnosed before 2 years of age. 7/8 children were in HIV care (6 on CTX; 3 on ART); 5 at Kalongo Hospital and 2 at Lira Kato HC. 3/5 children in care at Kalongo Hospital and 2/2 at Lira Kato HC were taken to their health facility on foot; 2/5 children were taken to Kalongo Hospital by bike. One child, aged 14 years (cared for by a grandmother) had not been seen in care for 10 months due to transport costs, having previously been seen at Kalongo Hospital.

## Discussion

There have been few population-based surveys in rural Africa reporting on general health, HIV-related knowledge, health-seeking behaviour and use of general and HIV health services. This survey was conducted in a remote area of northern Uganda which until 6 years ago, was at the centre of the rebel insurgency with the LRA; generalizability to other remote rural areas in Africa needs to take this into account. The survey was conducted before implementation of the Option B plus policy of ‘ART for life’ for pregnant women in Northern Uganda thus, although pMTCT services were de-centralised, this was not the case for general ART services. We surveyed all villages in the two parishes closest to Lira Kato Health Centre, comprising ~20 % of households and of the adult population in one sub-county. We included individuals most likely to use Lira Kato Health Centre for general healthcare; this could be a limitation of our findings if use of health services is higher in populations living close to a health centre than in those living further away.

Most indicators suggested lower household socio-economic status compared with the Uganda DHS results of 2011 for rural areas, demonstrating that this population is poorer than the average rural population in Uganda. However, it would not be unlike other populations living in hard-to-reach rural areas where comprehensive health services are not easily accessible close to where people live.

Most survey participants stated that they sought care locally for both fever and serious illness; however, around 1/4 people travelled the considerable distance to Kalongo hospital in the event of serious illness. Of note, there was a tendency to under-report the distance to Kalongo Hospital (approximately 28 km one way trip from Lapono Sub County).

Most study participants correctly identified most routes of HIV transmission. However, a significant minority of men and women thought HIV was spread by mosquitoes and other insects. This belief may have been due to outbreaks of yellow fever in this area, which is linked with mosquitoes. More than half of men and women were unaware that HIV can be transmitted trans-placentally during pregnancy, although most knew MTCT of HIV can occur intrapartum and through breastfeeding.

Reported rates of women and men who had ever been tested for HIV were slightly higher than reported for the mid-northern region in the Uganda AIDS Indicator Survey, 2011 (84 % vs. 81 % in women; 76 % vs. 63 % in men) [2]. The rate of testing in the last 12 months was particularly high in men (54  % vs. 39 %) in the Uganda AIDS Indicator Survey [2] and higher in men than in women; this may be due to safe male circumcision campaigns which had taken place at Lira Kato HC on three occasions in the 12 months before the survey and included HIV counselling and testing. Reported HIV prevalence (9 % in women; 6 % in men) was similar to anonymous testing data for the region (10 % in women and 6 % in men) [2], suggesting a high percentage of HIV-positive individuals were aware of their status and reported it correctly and/or that the area has high HIV-prevalence for the region.

Most women (96 %) had attended ANC at least once during the pregnancy leading to their most recent birth and more women attended ANC at least 4 times (as recommended by WHO) than is reported for rural areas of Uganda (52 % vs. 46 %) [2], although there remains room for improvement. The most common reason for not attending was the husband’s refusal to accompany their partner to clinic, which speaks to the need for more strategies to increase male involvement in ANC; this was also raised as a concern in the initial Lablite survey by healthcare workers [20]. Reasons for high antenatal attendance likely reflect a well-functioning ANC at the local HC III. However, among women who tested HIV positive in pregnancy, only around two thirds received drugs for pMTCT, as did similar proportions of their babies; it is unclear what happened to the remaining one third, or whether women reported this accurately. However, if accurate, this falls well below the target of 90 % uptake of pMTCT for pregnant women and babies set for Elimination of Mother to Child Transmission (EMTCT) [6].

We asked specific questions about testing and HIV care for children. Although in 26 households, participants reported that a child was infected with HIV; details of testing and care were provided for only 8 children. It is not clear whether reports of other HIV-positive children were accurate. In a further 30 households there were concerns reported that one or more children might be HIV-positive but they had not been tested, with a significant proportion not tested because of lack of knowledge about where to obtain testing. There were also reports of failure to return results of HIV tests for children. Estimates of the prevalence of HIV in children under 15 years in Uganda vary considerably; Uganda MOH based on the Spectrum model estimated that children constituted only 7 % of HIV-infected individuals in 2012 [21] whereas UNICEF figures suggest closer to 13 % [22]. These figures, based on 136 adults correspond to between 10 and 20 HIV-infected children, suggesting identification and enrolment into care of children may be low compared to adults. The concerns and lack of awareness around testing and the late diagnosis of HIV-infected children (6/8 children over 2 years) may reflect barriers and lack of knowledge among the community and healthcare workers.

Of the HIV-positive adults and children in care, 59 % were on ART. Despite the distance to ART services, this is similar to the 65 % derived from National figures for September 2013 (570,373 active ART patients: 883,736 HIV-infected persons in care) [21]. Some of the remainder who were being seen locally or were not in care may however have been in need of treatment; only half of those not on ART had had a CD4 test in the last year. In the consultative meeting with local village health teams (VHTs) prior to the survey we heard anecdotal reports that some of the community were unable to access care, particularly the old, children and the very sick, due to being unable to make the journey on foot.

## Conclusion

In summary, in this population survey in a remote poor rural area of Northern Uganda, despite difficulties in accessing ART treatment and care, HIV-infected individuals made considerable efforts to travel to receive services. In contrast most sought treatment for common conditions at local health facilities. Whereas antenatal services appeared good, only two thirds of women reported receiving pMTCT, perhaps reflecting poor partner support and possibly home births. HIV-infected children appeared to be under-accessing testing and referral for treatment. We infer that decentralization of the ART programme to primary care facilities would decrease travel time and transport costs and thus make care and treatment accessible to people not able to access centralized care; it might also improve follow-up especially in rural communities [23, 24].

## Additional files

Additional file 1:
**Is population based survey household questionnaire.** (PDF 643 kb) Additional file 2:
**Population based survey individual questionnaire.** (PDF 606 kb)

## Abbreviations

ANC: Antenatal care
ART: Antiretroviral therapy
CTX: Cotrimoxazole
EMTCT: Elimination of Mother to Child Transmission
GPS: Geographic positioning system
HC: Health centre
HCT: HIV counselling and testing
JCRC/REC: Joint Clinical Research Centre /Research Ethical Committee
LRA: Lord’s Resistance Army
PBS: Population based survey
PMTCT: Prevention of mother-to-child transmission
UDHS: Uganda demographic and health survey
UNCST: Uganda National Council for Science and Technology
VHTs: Village health teams
