# Supplementary material for: Population level usage of health services, and HIV testing and care, prior to decentralization of antiretroviral therapy in Agago District in rural Northern Uganda
Source: BMC Health Serv Res. 2015 Nov 28;15:527. doi: 10.1186/s12913-015-1194-4 (PMC4662831; doi:10.1186/s12913-015-1194-4)
Supplement: Additional file 1: — Is population based survey household questionnaire. (PDF 643 kb) [file 12913_2015_1194_MOESM1_ESM.pdf]

LABLITE PROJECT

POPULATION BASED SURVEY QUESTIONNAIRE

In Lira Kato sub-county, Agago District, Uganda.

Household questionnaire

Section A: Identification

A1	Name of interviewer<br>Nyiny ngat ma penyo peny	______________________	
A2	Interviewer code:<br>Nama pa ngat ma penyo peny	☐☐	**INT**
A4	Date of interview<br>Nino dwe me peny	☐☐☐☐☐☐	**DINT**
A5	Time interview begins:<br>Cawa ma lapeny cake kede	☐☐☐☐	**TINTBEG**
A6	Village number<br>Nama pi kabedo me caro	☐☐	**VNO**
A6	Household number<br>Nama me gang	☐☐☐	**HNO**
A7	Name of Participant<br>Nying lagam lapeny	______________________	**NAMEC**
A8	Participant's personal number<br>Nama pa lagam lapeny	☐☐	**PNO**

Section B: Socio-economic data

B1	Estimated date of birth<br>Biko nino nywal	day ☐☐ month ☐☐ year ☐☐☐☐	**DOB**
B2	Corrected age in years<br>Mwaka ni	☐☐	**AGER**

B3 Sex ☐ 1 = male, 2= female **SEX**

Nyako nyo awobi

B5 **Relationship of participant within household:**

In ibedo wat i gang eni:

☐ Yes = 1, No = 2 **RELHH**

If the participant is related to the head of household, recode relationship, and if not, SKIP to question B6.

Ka la gam peny tye wat pa won gang, coo kit wat te ka peke, kale iyi lapeny B6

RELATE

Head of household	☐ (1)	
Spouse	☐ (2)	
Child	☐ (3)	
Parent	☐ (4)	
Brother/sister	☐ (5)	
Grandchild	☐ (6)	
Nephew/niece	☐ (7)	
Paternal aunt /uncle	☐ (8)	
Maternal aunt/uncle	☐ (9)	
Paternal cousin	☐ (10)	
Maternal cousin	☐ (11)	
No relationship	☐ (12)	
Don't know	☐ (13)	
Other	☐ (14)	***specify***:_____________ _______________

Main house

Interviewer: for questions B6-14, please assess the roof, walls and number of main rooms (only ask if you are not sure of the answer from your observations). Circle or fill in the appropriate option.

Pi lapeny B6-14, timber nyi wii ot, koo ot, ki wel rum (peny keken ka itye ki akalakala ikom gin ma ineno). Lung ony pong ma rwate kwede.

B6 Type of roof? Ki t wii ot? **TROOF**

Thatch	☐ (1)	
Tile	☐ (2)	
Iron sheets	☐ (3)	
Plastic sheeting	☐ (4)	
Mixed iron/tile	☐ (5)	
Other	☐ (6)	***specify***:_____________ _______________

B7 Quality/state of the roof? Kit ber pa wii ot? **QROOF**

Good	☐ (1)
Fair	☐ (2)
Poor	☐ (3)

B8 Type of walls? Kit kor ot? **TWALL**

Mud and pole	☐ (1)
Bricks	☐ (2)

Concrete ☐ (3)

Mud/brick ☐ (4)

Brick/concrete ☐ (5)

Other ☐ (6) ***specify***:_____________ _______________

B9 How many people live in this house? Dano adii ma kwo i oti?

☐☐ Number **NUMHH**

Interviewer: please ask the respondent the following questions

Tim ber i peny lagam peny lapeny egini

House/property ownership

B10 Is the house owned by the household members living here? **OWNHSE**

Oti mono obedo pa dano ma kwo i?

☐ Yes = 1, No = 2

B11 Do household members own any income-generating houses or buildings (shop/kiosk, for example)? **OWNPROP**

Dano ma gi kwo i oti gi tye jami mogo ma kelo lim i cengi ma calo(labolle duka/kiosk)?

☐ Yes = 1, No = 2

Household amenities

B12 What is your main water source? **MAINWATER**

Poll pii mono onongo ki kwene?

River ☐ (1)

Well ☐ (2)

Protected spring	☐ (3)	
Borehole	☐ (4)	
Rain catchment	☐ (5)	
Water tap in house	☐ (6)	
Trench	☐ (7)	
Dam	☐ (8)	
Other	☐ (9)	***specify***:____________ ______________

B13 What alternative water sources do you use in addition to your main source or when main source is not available? **OTHWATER**

Yoo ango mukene ma onongo kwede pii ka pii wu obale nyo nongo ne odoko tek woko?

River	☐ (1)	
Well	☐ (2)	
Protected spring	☐ (3)	
Borehole	☐ (4)	
Rain catchment	☐ (5)	
Water tap in house	☐ (6)	
Trench	☐ (7)	
Dam	☐ (8)	
Other	☐ (9)	***specify***:____________

B14 Do you have a toilet? **TOILET**

Wu tye ki coron?

☐ Yes = 1, No = 2

If yes, what type? **TOILETTYPE**

Ka eyo, kit mene

Pit latrine ☐ (1)

Ventilated improved pit latrine ☐ (2)

Flush toilet ☐ (3)

Other ☐ (4) ***specify***:____________

B15 What is the predominant form of lighting the house at night? **LIGHT**

Ngo ma pol kare otiyo kwede me menyo ot i dyewor?

Electricity (HEP, Generator, Solar) ☐ (1)

Paraffin lantern ☐ (2)

Wax candle ☐ (3)

Paraffin candle ☐ (4)

Other ☐ (5) ***specify***:____________

Household items

I am going to read to you a list of things used in a home. Can you tell me which of them you have and if they are in working order, or if you had and sold in the past year, or if you owned more than a year ago? Abi kwani jami magi ma gitiyo kwede i ot .waca mene ma tye dok tiyo,nyo ibedo tye kwede ento icato imwaka mukato,nyo ibedo kwede con

Item	Owned and in working order	Sold in the past year	Not owned now but it was owned more than a year ago	Don't own (Nil)	Variable Name

	(1)	(2)	(3)	(4)	
Plastic jerry can					**IHH1**
Pot for keeping drinking water					**IHH2**
Dish drying rack					**IHH3**
Bed					**IHH4**
Mattress (foam)					**IHH5**
Mosquito net					**IHH6**
Radio/radio cassette player					**IHH7**
Television					**IHH8**
Sewing machine					**IHH9**
Bicycle					**IHH10**
Motorcycle					**IHH11**
Motor car or truck					**IHH12**
Cell phone					**IHH13**
Breakable cups					**IHH14**
Weighing scale (for produce)					**IHH15**
Paraffin stove (cooking)					**IHH16**
Charcoal stove (cooking)					**IHH17**
Gas stove (cooking)					**IHH18**
Paraffin lamp					**IHH19**

Land

B17 Does the land on which this household lives belong to the household, or is it rented or borrowed? **OWNLD**

Ngom ma obed iye ni meg wu,opango apanga onyo olego alega?

Own land ☐ (1)

Rented ☐ (2)

Borrowed ☐ (3)

Not sure ☐ (4)

B18 What is your source of livelihood? **LIVELHD**

Yoo ango mu okwo kwede?

(select all that apply)

Livestock farmer	☐ (1)	
Craftsperson	☐ (2)	
Mechanic	☐ (3)	
Brick maker	☐ (4)	
Subsistence crop/ vegetable cultivator	☐ (5)	
Cash crop grower	☐ (6)	
Civil servant	☐ (7)	
Alcohol brewing	☐ (8)	
Tailor	☐ (9)	
Miller	☐ (10)	
Petty / retail business	☐ (11)	
Boda boda cyclist	☐ (12)	
Other	☐ (13)	***specify***:_____________ _______________

Section C: Children

C1 How many children (yours or others) under 15 years are living with you?

Lutino adii ma megi nyo pe megi ma mwaka gi pe oo 15 ma i kwo kwede gi?

☐☐Number **NUMCH**

C2 How many are your biological children?

Adii ma in inywalo anywala?

☐☐ Number **OWNCH**

C3 Have any children had a positive HIV test? **TESTPOS**

Ikin gi lutini moo ki pimo ki nongo ni tye ki kwidi two jonyo?

☐ Yes = 1, No = 2, Never tested=3

If YES, number of children? Ka eyo, lutino adii ?

☐☐ Number **CHILDPOS**

C4 Do you think that any children in your household who haven't been tested may have HIV infection? **WORPOS**

I tye ki lworo ni ikin lutini ma pe gipimo moo room bedo ki kwidi me two jonyo?

☐ Yes = 1, No = 2

If NO STOP, finish interview (**Ka ku, gik kany, lapeny otum**)

If yes, how many children? ☐☐ Number **NUMWOR**

Ka eyo, lutino adii?

If respondent answers YES to C4, then ask the following questions C5-C6

Ka lagam peny oye ni eyo i C4, ci peny lapeny magi C5-C6

C5 What are their ages? (Begin with youngest child)

Mwaki gi adii? (cak ki matidi loyo)

Age ☐☐ Years **AGE1**

Age ☐☐ Years **AGE2**

Age ☐☐ Years **AGE3**

Age ☐☐ Years **AGE4**

Age ☐☐ Years **AGE5**

C6 Why have they not been tested? **REASCNT**

Pi ngong pe gi pimo gi?

- I don't want to know their HIV results ☐ (1)
- I fear having them tested for HIV ☐ (2)
- Counseling offices are too far ☐ (3)
- I'm not interested ☐ (4)
- I don't know where to take them ☐ (5)

For any child who has had a positive HIV test: (compete below for each child beginning with the youngest)

Pi latin mo keken ma ki pimo ki nongo tye ki kwidi me two jonyo: (pong peny pi latin ace acel cake ki ma tidi loyo

Child 1

C7 How old is he/she now (youngest HIV-positive child)? (Begin with youngest child)

En tye mwaka adii kom bedi? (cak ki latin ma tye ki kwidi two jonyo ma tidi loyo)

Age ☐☐ Years **CHILD1**

C8 Who is his/her primary carer? **PCARER1**

Angaa ma gwoke?

- Father ☐ (1)
- Mother ☐ (2)

Grandmother	☐ (3)	
Grandfather	☐ (4)	
Aunt	☐ (5)	
Uncle	☐ (6)	
Brother/sister	☐ (7)	
Other	☐ (8)	***specify***:_____________ _______________

C9 How old was he/she at diagnosis?

Mwaka ne onongo tye adii ikare ma gi pimo gi nongo tye ki two?

Age ☐☐ Years **AGEDIAG1**

C10 Has he/she ever received septrin? **EVERCOT1**

En dong ogamo yat septrin?

☐ Yes = 1, No = 2

If YES, is he/she receiving septrin now? **NOWCOT1**

Ka eyo, en pud gamo yat septrin?

☐ Yes = 1, No = 2

C11 Has he/she ever received ARVs? **EVERART1**

En dong ogamo yat lagin?

☐ Yes = 1, No = 2

If YES, is he/she receiving ARVs now? **NOWART1**

Ka eyo, en pud gamo yat lagin?

☐ Yes = 1, No = 2

Questions if child is not on septrin or ARVs now

Lapeny ka latin peke ka mwunyu septrin onyo lajin kom kare ni

C12 When did the child last attend a health facility for HIV care/treatment?

A wene ma latin ogik kwede cento iot yat pi nongo kony me yat?

☐☐ month **MTHCARE1**

☐☐☐☐ year **YRCARE1**

C13 Which facility did he/she last attend? **CAREFAC1**

Ot yat mene ma en oceto i me agiki?

Kalongo mission hospital	☐ (1)	
Private local clinic	☐ (2)	
Patongo HC IV	☐ (3)	
Lira Kato HC III	☐ (4)	
Public clinic	☐ (5)	
Other	☐ (6)	***specify***:____________ ______________

C14 How far is the facility from home (distance in kilometres)?

Boo pa ot yat tye lakwene gi gang (i kilometa)?

☐☐☐km **DISTC1**

C15 How long (hours, minutes) did it take to take him/her to the facility, receive medical care, and return home?

Tero kare ma rom mene (cawa, dakika) ki in me oo iot yat, neno darta, gamo yat, ki dok gang?

☐☐ hours HRSC1

☐☐ minutes MINSC1

C16 What mode of transport was used to take the child to the facility? **TRHFAC1**

Gin wot ango ma itiyo kwede me cwalo latini i ot yat?

Own bicycle ☐ (1)

Bicycle taxi ☐ (2)

Own motor bike ☐ (3)

Motorbike taxi ☐ (4)

Bus ☐ (5)

Special hire taxi/cab ☐ (6)

Borrowed bicycle ☐ (7)

On foot ☐ (8)

Other ☐ (9) ***specify***:____________ ______________

C17 Why is the child not on treatment? **REASTRT1**

Pi ngo latini pe tye ka nongo kony me yat?

Cost of drugs ☐ (1)

Transport cost ☐ (2)

Other ☐ (3) ***specify***:____________ ______________

Questions if child is on septrin or ARVs now

Lapeny ka latin tye ka mwunyu septrin onyo lajin kom kare ni

C18 Which facility does he/she usually attend for treatment? **FACNOW1**

Ot yat mene ma en pol kare ceto iye ka nongo kony me yat?

Lira kato HC III ☐ (1)

Patongo HC IV ☐ (2)

Kalongo mission hospital ☐ (3)

Other ☐ (4) *specify*:____________ ______________

C19 Who usually takes him/her? **FNCARE1**

Angaa ma pol kare tero en?

Father ☐ (1)

Mother ☐ (2)

Grandmother ☐ (3)

Grandfather ☐ (4)

Aunt ☐ (5)

Uncle ☐ (6)

Brother/sister ☐ (7)

None ☐ (8)

Other ☐ (9) *specify*:____________ ______________

C20 How far is the facility from home (distance in kilometres)?

Ot yat bor rom mene ki gang (bore i kilometa)?

☐☐☐ km **DISTFN1**

C21 How long (hours, minutes) did it take to take him/her to the facility, receive medical care, and return home?

Tero kare ma rom mene (cawa, dakika) ki in me oo iot yat, neno darta, gamo yat, ki dok gang?

☐☐ hours **HRFN1**

☐☐ minutes **MINFN1**

C22 What mode of transport is usually used to take the child to the facility? **TRFNOW1**

Gin wot ango ma itiyo kwede me cwalo latini i ot yat?

Own bicycle	☐ (1)
Bicycle taxi	☐ (2)
Own motor bike	☐ (3)
Motorbike taxi	☐ (4)
Bus	☐ (5)
Special hire taxi/cab	☐ (6)
Borrowed bicycle	☐ (7)
On foot	☐ (8)
Other	☐ (9) ***specify***:____________ ______________

C23 At the child's last visit to this health facility did you have to pay fees to receive care/treatment?

I culu pi kony me yat ikare ma itero latini me agiki i ot man?

☐ Yes = 1, No = 2 **CHFEES1**

If yes, how much did you pay in Shillings? Ka eyo, iculu ciling adi?

☐☐☐☐☐☐ Shs. **CHCOST1**

C24 In the last year, has the child missed any appointments for HIV care/treatment?

Imwaka ma okato ni, latini okeng ceto i ot yat i nino ma giciko me nong kony me yat?

☐ Yes = 1, No = 2 **CMISART1**

If YES, how many times? Ka eyo, tyen adii?

☐☐ Number of times **CTIMISA1**

C25 In the last year has anyone been to a health facility (with or without the child) to collect septrin for the child and been unable to?

I mwaka mukato ni ngati oceto i ot yat(ki latin onyo labong latin) ka gamo septrin , ento pe ogamo?

☐ Yes = 1, No = 2 **CUNPCOT1**

If YES (unable to pick up drugs), what is the main reason you've been unable to collect septrin?

Ka eyo, (pe ogamo yat), pi tyen lok ango ma oweko en pe ogamo yat septrin

CREASCOTP1

Drugs out of stock	☐ (1)	
ART clinic was closed	☐ (2)	
Referred to another facility to change treatment	☐ (3)	
No health workers at facility	☐ (4)	
Other	☐ (5)	***specify***:_____________

C26 In the last year has anyone been to a health facility (with or without the child) to collect ARVs for the child and been unable to?

I mwaka mukato ni ngati oceto i ot yat(ki latin onyo labong latin) ka gamo lagin , ento pe ogamo?

☐ Yes = 1, No = 2 **CUNPART1**

If YES (unable to pick up drugs), what is the main reason you've been unable to collect ARVs?

Ka eyo (pe ogamo yat), pi tyen lok ango ma oweko en pe ogamo yat lagin

CREASARTP1

Drugs out of stock	☐ (1)	
ART clinic was closed	☐ (2)	
Referred to another facility to change treatment	☐ (3)	
No health workers at facility	☐ (4)	
Other	☐ (5)	***specify***:_____________

Child 2

C27 How old is he/she now (youngest HIV-positive child)? (Begin with youngest child)

En tye mwaka adii kom bedi? (cak ki latin ma tye ki kwidi two jonyo ma tidi loyo)

Age ☐☐ Years **CHILD2**

C28 Who is his/her primary carer? **PCARER2**

Angaa ma gwoke?

Father ☐ (1)

Mother ☐ (2)

Grandmother ☐ (3)

Grandfather ☐ (4)

Aunt ☐ (5)

Uncle ☐ (6)

Brother/sister ☐ (7)

Other ☐ (8) ***specify***:____________ ______________

C29 How old was he/she at diagnosis?

Mwaka ne onongo tye adii ikare ma gi pimo gi nongo tye ki two?

Age ☐☐ Years **AGEDIAG2**

C30 Has he/she ever received septrin? **EVERCOT2**

En dong ogamo yat septrin?

☐ Yes = 1, No = 2

If YES, is he/she receiving septrin now? **NOWCOT2**

Ka eyo, en pud gamo yat septrin?

☐ Yes = 1, No = 2

C31 Has he/she ever received ARVs? **EVERART2**

En dong ogamo yat lagin?

☐ Yes = 1, No = 2

If YES, is he/she receiving ARVs now? **NOWART2**

Ka eyo, en pud gamo yat lagin?

☐ Yes = 1, No = 2

Questions if child is not on septrin or ARVs now

Lapeny ka latin peke ka mwunyu septrin onyo lajin kom kare ni

C32 When did the child last attend a health facility for HIV care/treatment?

A wene ma latin ogik kwede cento iot yat pi nongo kony me yat?

☐☐ month **MTHCARE2**

☐☐☐☐ year **YRCARE1**

C33 Which facility did he/she last attend? **CAREFAC2**

Ot yat mene ma en oceto i me agiki?

Kalongo mission hospital	☐ (1)
Private local clinic	☐ (2)
Patongo HC IV	☐ (3)
Lira Kato HC III	☐ (4)
Public clinic	☐ (5)
Other	☐ (6) ***specify***:_____________ _______________

C34 How far is the facility from home (distance in kilometres)?

Boo pa ot yat tye lakwene gi gang (i kilometa)?

☐☐☐km **DISTC2**

C35 How long (hours, minutes) did it take to take him/her to the facility, receive medical care, and return home?

Tero kare ma rom mene (cawa, dakika) ki in me oo iot yat, neno darta, gamo yat, ki dok gang?

☐☐ hours **HRSC2**

☐☐ minutes **MINSC2**

C36 What mode of transport was used to take the child to the facility? **TRHFAC2**

Gin wot ango ma itiyo kwede me cwalo latini i ot yat?

Own bicycle ☐ (1)

Bicycle taxi ☐ (2)

Own motor bike ☐ (3)

Motorbike taxi ☐ (4)

Bus ☐ (5)

Special hire taxi/cab ☐ (6)

Borrowed bicycle ☐ (7)

On foot ☐ (8)

Other ☐ (9) ***specify***:_____________ _______________

C37 Why is the child not on treatment? **REASTRT2**

Pi ngo latini pe tye ka nongo kony me yat?

Cost of drugs ☐ (1)

Transport cost ☐ (2)

Other ☐ (3) ***specify***:_____________ _______________

Questions if child is on septrin or ARVs now

Lapeny ka latin tye ka mwunyu septrin onyo lajin kom kare ni

C38 Which facility does he/she usually attend for treatment? **FACNOW2**

Ot yat mene ma en pol kare ceto iye ka nongo kony me yat?

Lira kato HC III ☐ (1)

Patongo HC IV ☐ (2)

Kalongo mission hospital ☐ (3)

Other ☐ (4) ***specify***:_____________ _______________

C39 Who usually takes him/her? **FNCARE2**

Angaa ma pol kare tero en?

Father ☐ (1)

Mother ☐ (2)

Grandmother ☐ (3)

Grandfather ☐ (4)

Aunt ☐ (5)

Uncle ☐ (6)

Brother/sister ☐ (7)

None ☐ (8)

Other ☐ (9) ***specify***:_____________ _______________

C40 How far is the facility from home (distance in kilometres)?

Ot yat bor rom mene ki gang (bore i kilometa)?

☐☐☐ km **DISTFN2**

C41 How long (hours, minutes) did it take to take him/her to the facility, receive medical care, and return home?

Tero kare ma rom mene (cawa, dakika) ki in me oo iot yat, neno darta, gamo yat, ki dok gang?

☐☐ hours **HRFN2**

☐☐ minutes **MINFN2**

C42 What mode of transport is usually used to take the child to the facility? **TRFNOW2**

Gin wot ango ma itiyo kwede me cwalo latini i ot yat?

Own bicycle	☐ (1)	
Bicycle taxi	☐ (2)	
Own motor bike	☐ (3)	
Motorbike taxi	☐ (4)	
Bus	☐ (5)	
Special hire taxi/cab	☐ (6)	
Borrowed bicycle	☐ (7)	
On foot	☐ (8)	
Other	☐ (9)	***specify***:____________ ______________

C43 At the child's last visit to this health facility did you have to pay fees to receive care/treatment?

I culu pi kony me yat ikare ma itero latini me agiki i ot man?

☐ Yes = 1, No = 2 **CHFEES2**

If yes, how much did you pay in Shillings? Ka eyo, iculu ciling adi?

☐☐☐☐☐☐ Shs. **CHCOST2**

C44 In the last year, has the child missed any appointments for HIV care/treatment?

Imwaka ma okato ni, latini okeng ceto i ot yat i nino ma giciko me nong kony me yat?

☐ Yes = 1, No = 2 **CMISART2**

If YES, how many times? Ka eyo, tyen adii?

☐☐ Number of times **CTIMISA2**

C45 In the last year has anyone been to a health facility (with or without the child) to collect septrin for the child and been unable to?

I mwaka mukato ni ngati oceto i ot yat(ki latin onyo labong latin) ka gamo septrin , ento pe ogamo?

☐ Yes = 1, No = 2 **CUNPCOT2**

If YES (unable to pick up drugs), what is the main reason you've been unable to collect septrin?

Ka eyo, (pe ogamo yat), pi tyen lok ango ma oweko en pe ogamo yat septrin

CREASCOTP2

Drugs out of stock	☐ (1)
ART clinic was closed	☐ (2)
Referred to another facility to change treatment	☐ (3)
No health workers at facility	☐ (4)
Other	☐ (5) *specify*:____________

C46 In the last year has anyone been to a health facility (with or without the child) to collect ARVs for the child and been unable to?

I mwaka mukato ni ngati oceto i ot yat(ki latin onyo labong latin) ka gamo lagin , ento pe ogamo?

☐ Yes = 1, No = 2 **CUNPART2**

If YES (unable to pick up drugs), what is the main reason you've been unable to collect ARVs?

Ka eyo (pe ogamo yat), pi tyen lok ango ma oweko en pe ogamo yat lagin

CREASARTP2

Drugs out of stock	☐ (1)
ART clinic was closed	☐ (2)
Referred to another facility to change treatment	☐ (3)
No health workers at facility	☐ (4)
Other	☐ (5) *specify*:____________

Child 3

C47 How old is he/she now (youngest HIV-positive child)? (Begin with youngest child)

En tye mwaka adii kom bedi? (cak ki latin ma tye ki kwidi two jonyo ma tidi loyo)

Age ☐☐ Years **CHILD3**

C48 Who is his/her primary carer? **PCARER3**

Angaa ma gwoke?

Father	☐ (1)	
Mother	☐ (2)	
Grandmother	☐ (3)	
Grandfather	☐ (4)	
Aunt	☐ (5)	
Uncle	☐ (6)	
Brother/sister	☐ (7)	
Other	☐ (8)	***specify***:____________ ______________

C49 How old was he/she at diagnosis?

Mwaka ne onongo tye adii ikare ma gi pimo gi nongo tye ki two?

Age ☐☐ Years **AGEDIAG3**

C50 Has he/she ever received septrin? **EVERCOT3**

En dong ogamo yat septrin?

☐ Yes = 1, No = 2

If YES, is he/she receiving septrin now? **NOWCOT3**

Ka eyo, en pud gamo yat septrin?

☐ Yes = 1, No = 2

C51 Has he/she ever received ARVs? **EVERART3**

En dong ogamo yat lagin?

☐ Yes = 1, No = 2

If YES, is he/she receiving ARVs now? **NOWART3**

Ka eyo, en pud gamo yat lagin?

☐ Yes = 1, No = 2

Questions if child is not on septrin or ARVs now

Lapeny ka latin peke ka mwunyu septrin onyo lajin kom kare ni

C52 When did the child last attend a health facility for HIV care/treatment?

A wene ma latin ogik kwede cento iot yat pi nongo kony me yat?

☐☐ month **MTHCARE3**

☐☐☐☐ year **YRCARE3**

C53 Which facility did he/she last attend? **CAREFAC3**

Ot yat mene ma en oceto i me agiki?

Kalongo mission hospital ☐ (1)

Private local clinic ☐ (2)

Patongo HC IV ☐ (3)

Lira Kato HC III ☐ (4)

Public clinic ☐ (5)

Other ☐ (6) ***specify***:_____________ _______________

C54 How far is the facility from home (distance in kilometres)?

Boo pa ot yat tye lakwene gi gang (i kilometa)?

☐☐☐km **DISTC3**

C55 How long (hours, minutes) did it take to take him/her to the facility, receive medical care, and return home?

Tero kare ma rom mene (cawa, dakika) ki in me oo iot yat, neno darta, gamo yat, ki dok gang?

☐☐ hours **HRSC3**

☐☐ minutes **MINSC3**

C56 What mode of transport was used to take the child to the facility? **TRHFAC3**

Gin wot ango ma itiyo kwede me cwalo latini i ot yat?

Own bicycle ☐ (1)

Bicycle taxi ☐ (2)

Own motor bike ☐ (3)

Motorbike taxi ☐ (4)

Bus ☐ (5)

Special hire taxi/cab ☐ (6)

Borrowed bicycle ☐ (7)

On foot ☐ (8)

Other ☐ (9) ***specify***:____________ ______________

C57 Why is the child not on treatment? **REASTRT3**

Pi ngo latini pe tye ka nongo kony me yat?

Cost of drugs ☐ (1)

Transport cost ☐ (2)

Other ☐ (3) ***specify***:____________ ______________

Questions if child is on septrin or ARVs now

Lapeny ka latin tye ka mwunyu septrin onyo lajin kom kare ni

C58 Which facility does he/she usually attend for treatment? **FACNOW3**

Ot yat mene ma en pol kare ceto iye ka nongo kony me yat?

Lira kato HC III (1)

Patongo HC IV (2)

Kalongo mission hospital (3)

Other (4) ***specify***:_____________ _______________

C59 Who usually takes him/her? **FNCARE3**

Angaa ma pol kare tero en?

Father (1)

Mother (2)

Grandmother (3)

Grandfather (4)

Aunt (5)

Uncle (6)

Brother/sister (7)

None (8)

Other (9) ***specify***:_____________ _______________

C60 How far is the facility from home (distance in kilometres)?

Ot yat bor rom mene ki gang (bore i kilometa)?

km **DISTFN3**

C61 How long (hours, minutes) did it take to take him/her to the facility, receive medical care, and return home?

Tero kare ma rom mene (cawa, dakika) ki in me oo iot yat, neno darta, gamo yat, ki dok gang?

hours **HRFN3**

minutes **MINFN3**

C62 What mode of transport is usually used to take the child to the facility? **TRFNOW3**

Gin wot ango ma itiyo kwede me cwalo latini i ot yat?

Own bicycle	☐ (1)	
Bicycle taxi	☐ (2)	
Own motor bike	☐ (3)	
Motorbike taxi	☐ (4)	
Bus	☐ (5)	
Special hire taxi/cab	☐ (6)	
Borrowed bicycle	☐ (7)	
On foot	☐ (8)	
Other	☐ (9)	***specify***:____________ ______________

C63 At the child's last visit to this health facility did you have to pay fees to receive care/treatment?

I culu pi kony me yat ikare ma itero latini me agiki i ot man?

☐ Yes = 1, No = 2 **CHFEES3**

If yes, how much did you pay in Shillings? Ka eyo, iculu ciling adi?

☐☐☐☐☐☐ Shs. **CHCOST3**

C64 In the last year, has the child missed any appointments for HIV care/treatment?

Imwaka ma okato ni, latini okeng ceto i ot yat i nino ma giciko me nong kony me yat?

☐ Yes = 1, No = 2 **CMISART3**

If YES, how many times? Ka eyo, tyen adii?

☐☐ Number of times **CTIMISA3**

C65 In the last year has anyone been to a health facility (with or without the child) to collect septrin for the child and been unable to?

I mwaka mukato ni ngati oceto i ot yat(ki latin onyo labong latin) ka gamo septrin , ento pe ogamo?

☐ Yes = 1, No = 2 **CUNPCOT3**

If YES (unable to pick up drugs), what is the main reason you've been unable to collect septrin?

Ka eyo, (pe ogamo yat), pi tyen lok ango ma oweko en pe ogamo yat septrin

CREASCOTP3

Drugs out of stock ☐ (1)

ART clinic was closed ☐ (2)

Referred to another facility to change treatment ☐ (3)

No health workers at facility ☐ (4)

Other ☐ (5) ***specify***:_____________

C66 In the last year has anyone been to a health facility (with or without the child) to collect ARVs for the child and been unable to?

I mwaka mukato ni ngati oceto i ot yat(ki latin onyo labong latin) ka gamo lagin , ento pe ogamo?

☐ Yes = 1, No = 2 **CUNPART3**

If YES (unable to pick up drugs), what is the main reason you've been unable to collect ARVs?

Ka eyo (pe ogamo yat), pi tyen lok ango ma oweko en pe ogamo yat lagin

CREASARTP3

Drugs out of stock ☐ (1)

ART clinic was closed ☐ (2)

Referred to another facility to change treatment ☐ (3)

No health workers at facility ☐ (4)

Other ☐ (5) ***specify***:_____________

Child 4

C67 How old is he/she now (youngest HIV-positive child)? (Begin with youngest child)

En tye mwaka adii kom bedi? (cak ki latin ma tye ki kwidi two jonyo ma tidi loyo)

Age ☐☐ Years **CHILD4**

C68 Who is his/her primary carer? **PCARER4**

Angaa ma gwoke?

Father	☐ (1)	
Mother	☐ (2)	
Grandmother	☐ (3)	
Grandfather	☐ (4)	
Aunt	☐ (5)	
Uncle	☐ (6)	
Brother/sister	☐ (7)	
Other	☐ (8)	***specify***:_____________ _______________

C69 How old was he/she at diagnosis?

Mwaka ne onongo tye adii ikare ma gi pimo gi nongo tye ki two?

Age ☐☐ Years **AGEDIAG4**

C70 Has he/she ever received septrin? **EVERCOT4**

En dong ogamo yat septrin?

☐ Yes = 1, No = 2

If YES, is he/she receiving septrin now? **NOWCOT4**

Ka eyo, en pud gamo yat septrin?

☐ Yes = 1, No = 2

C71 Has he/she ever received ARVs? **EVERART4**

En dong ogamo yat lagin?

☐ Yes = 1, No = 2

If YES, is he/she receiving ARVs now? **NOWART4**

Ka eyo, en pud gamo yat lagin?

☐ Yes = 1, No = 2

Questions if child is not on septrin or ARVs now

Lapeny ka latin peke ka mwunyu septrin onyo lajin kom kare ni

C72 When did the child last attend a health facility for HIV care/treatment?

A wene ma latin ogik kwede cento iot yat pi nongo kony me yat?

☐☐ month **MTHCARE4**

☐☐☐☐ year **YRCARE4**

C73 Which facility did he/she last attend? **CAREFAC4**

Ot yat mene ma en oceto i me agiki?

Kalongo mission hospital ☐ (1)

Private local clinic ☐ (2)

Patongo HC IV ☐ (3)

Lira Kato HC III ☐ (4)

Public clinic ☐ (5)

Other ☐ (6) ***specify***:_____________ _______________

C74 How far is the facility from home (distance in kilometres)?

Boo pa ot yat tye lakwene gi gang (i kilometa)?

☐☐☐ km DISTC4

C75 How long (hours, minutes) did it take to take him/her to the facility, receive medical care, and return home?

Tero kare ma rom mene (cawa, dakika) ki in me oo iot yat, neno darta, gamo yat, ki dok gang?

☐☐ hours **HRSC4**

☐☐ minutes **MINSC4**

C76 What mode of transport was used to take the child to the facility? **TRHFAC4**

Gin wot ango ma itiyo kwede me cwalo latini i ot yat?

Own bicycle	☐ (1)	
Bicycle taxi	☐ (2)	
Own motor bike	☐ (3)	
Motorbike taxi	☐ (4)	
Bus	☐ (5)	
Special hire taxi/cab	☐ (6)	
Borrowed bicycle	☐ (7)	
On foot	☐ (8)	
Other	☐ (9)	***specify***:_____________ _______________

C77 Why is the child not on treatment? **REASTRT4**

Pi ngo latini pe tye ka nongo kony me yat?

Cost of drugs	☐ (1)	
Transport cost	☐ (2)	
Other	☐ (3)	***specify***:_____________ _______________

Questions if child is on septrin or ARVs now

Lapeny ka latin tye ka mwunyu septrin onyo lajin kom kare ni

C78 Which facility does he/she usually attend for treatment? **FACNOW4**

Ot yat mene ma en pol kare ceto iye ka nongo kony me yat?

Lira kato HC III	☐ (1)	
Patongo HC IV	☐ (2)	
Kalongo mission hospital	☐ (3)	
Other	☐ (4)	***specify***:____________ ______________

C79 Who usually takes him/her? **FNCARE4**

Angaa ma pol kare tero en?

Father	☐ (1)	
Mother	☐ (2)	
Grandmother	☐ (3)	
Grandfather	☐ (4)	
Aunt	☐ (5)	
Uncle	☐ (6)	
Brother/sister	☐ (7)	
None	☐ (8)	
Other	☐ (9)	***specify***:____________ ______________

C80 How far is the facility from home (distance in kilometres)?

Ot yat bor rom mene ki gang (bore i kilometa)?

☐☐☐km **DISTFN4**

C81 How long (hours, minutes) did it take to take him/her to the facility, receive medical care, and return home?

Tero kare ma rom mene (cawa, dakika) ki in me oo iot yat, neno darta, gamo yat, ki dok gang?

☐☐ hours HRFN4

☐☐ minutes MINFN4

C82 What mode of transport is usually used to take the child to the facility? **TRFNOW4**

Gin wot ango ma itiyo kwede me cwalo latini i ot yat?

Own bicycle	☐ (1)	
Bicycle taxi	☐ (2)	
Own motor bike	☐ (3)	
Motorbike taxi	☐ (4)	
Bus	☐ (5)	
Special hire taxi/cab	☐ (6)	
Borrowed bicycle	☐ (7)	
On foot	☐ (8)	
Other	☐ (9)	***specify***:____________ ______________

C83 At the child's last visit to this health facility did you have to pay fees to receive care/treatment?

I culu pi kony me yat ikare ma itero latini me agiki i ot man?

☐ Yes = 1, No = 2 **CHFEES4**

If yes, how much did you pay in Shillings? Ka eyo, iculu ciling adi?

☐☐☐☐☐☐ Shs. **CHCOST4**

C84 In the last year, has the child missed any appointments for HIV care/treatment?

Imwaka ma okato ni, latini okeng ceto i ot yat i nino ma giciko me nong kony me yat?

☐ Yes = 1, No = 2 **CMISART4**

If YES, how many times? Ka eyo, tyen adii?

☐☐ Number of times **CTIMISA4**

C85 In the last year has anyone been to a health facility (with or without the child) to collect septrin for the child and been unable to?

I mwaka mukato ni ngati oceto i ot yat(ki latin onyo labong latin) ka gamo septrin , ento pe ogamo?

☐ Yes = 1, No = 2 **CUNPCOT4**

If YES (unable to pick up drugs), what is the main reason you've been unable to collect septrin?

Ka eyo, (pe ogamo yat), pi tyen lok ango ma oweko en pe ogamo yat septrin

CREASCOTP4

Drugs out of stock ☐ (1)

ART clinic was closed ☐ (2)

Referred to another facility to change treatment ☐ (3)

No health workers at facility ☐ (4)

Other ☐ (5) ***specify***:_____________

C86 In the last year has anyone been to a health facility (with or without the child) to collect ARVs for the child and been unable to?

I mwaka mukato ni ngati oceto i ot yat(ki latin onyo labong latin) ka gamo lagin , ento pe ogamo?

☐ Yes = 1, No = 2 **CUNPART4**

If YES (unable to pick up drugs), what is the main reason you've been unable to collect ARVs?

Ka eyo (pe ogamo yat), pi tyen lok ango ma oweko en pe ogamo yat lagin

CREASARTP4

Drugs out of stock ☐ (1)

ART clinic was closed ☐ (2)

Referred to another facility to change treatment ☐ (3)

No health workers at facility ☐ (4)

Other ☐ (5) ***specify***:____________

Child 5

C87 How old is he/she now (youngest HIV-positive child)? (Begin with youngest child)

En tye mwaka adii kom bedi? (cak ki latin ma tye ki kwidi two jonyo ma tidi loyo)

Age ☐☐ Years **CHILD5**

C88 Who is his/her primary carer? **PCARER5**

Angaa ma gwoke?

Father	☐ (1)	
Mother	☐ (2)	
Grandmother	☐ (3)	
Grandfather	☐ (4)	
Aunt	☐ (5)	
Uncle	☐ (6)	
Brother/sister	☐ (7)	
Other	☐ (8)	***specify***:____________ ______________

C89 How old was he/she at diagnosis?

Mwaka ne onongo tye adii ikare ma gi pimo gi nongo tye ki two?

Age ☐☐ Years **AGEDIAG5**

C90 Has he/she ever received septrin? **EVERCOT5**

En dong ogamo yat septrin?

☐ Yes = 1, No = 2

If YES, is he/she receiving septrin now? **NOWCOT5**

Ka eyo, en pud gamo yat septrin?

☐ Yes = 1, No = 2

C91 Has he/she ever received ARVs? **EVERART5**

En dong ogamo yat lagin?

☐ Yes = 1, No = 2

If YES, is he/she receiving ARVs now? **NOWART5**

Ka eyo, en pud gamo yat lagin?

☐ Yes = 1, No = 2

Questions if child is not on septrin or ARVs now

Lapeny ka latin peke ka mwunyu septrin onyo lajin kom kare ni

C92 When did the child last attend a health facility for HIV care/treatment?

A wene ma latin ogik kwede cento iot yat pi nongo kony me yat?

☐☐ month **MTHCARE5**

☐☐☐☐ year **YRCARE5**

C93 Which facility did he/she last attend? **CAREFAC5**

Ot yat mene ma en oceto i me agiki?

Kalongo mission hospital ☐ (1)

Private local clinic ☐ (2)

Patongo HC IV ☐ (3)

Lira Kato HC III ☐ (4)

Public clinic	☐ (5)		
Other	☐ (6)	***specify***:_____________ _______________	

C94 How far is the facility from home (distance in kilometres)?

Boo pa ot yat tye lakwene gi gang (i kilometa)?

☐☐☐ km **DISTC5**

C95 How long (hours, minutes) did it take to take him/her to the facility, receive medical care, and return home?

Tero kare ma rom mene (cawa, dakika) ki in me oo iot yat, neno darta, gamo yat, ki dok gang?

☐☐ hours **HRSC5**

☐☐ minutes **MINSC5**

C96 What mode of transport was used to take the child to the facility? **TRHFAC5**

Gin wot ango ma itiyo kwede me cwalo latini i ot yat?

Own bicycle	☐ (1)	
Bicycle taxi	☐ (2)	
Own motor bike	☐ (3)	
Motorbike taxi	☐ (4)	
Bus	☐ (5)	
Special hire taxi/cab	☐ (6)	
Borrowed bicycle	☐ (7)	
On foot	☐ (8)	
Other	☐ (9)	***specify***:_____________ _______________

C97 Why is the child not on treatment? **REASTRT5**

Pi ngo latini pe tye ka nongo kony me yat?

Cost of drugs ☐ (1)

Transport cost ☐ (2)

Other ☐ (3) ***specify***:_____________ _______________

Questions if child is on septrin or ARVs now

Lapeny ka latin tye ka mwunyu septrin onyo lajin kom kare ni

C98 Which facility does he/she usually attend for treatment? **FACNOW5**

Ot yat mene ma en pol kare ceto iye ka nongo kony me yat?

Lira kato HC III ☐ (1)

Patongo HC IV ☐ (2)

Kalongo mission hospital ☐ (3)

Other ☐ (4) ***specify***:_____________ _______________

C99 Who usually takes him/her? **FNCARE5**

Angaa ma pol kare tero en?

Father ☐ (1)

Mother ☐ (2)

Grandmother ☐ (3)

Grandfather ☐ (4)

Aunt ☐ (5)

Uncle ☐ (6)

Brother/sister ☐ (7)

None ☐ (8)

Other ☐ (9) ***specify***:_____________ _______________

C100 How far is the facility from home (distance in kilometres)?

Ot yat bor rom mene ki gang (bore i kilometa)?

[ ][ ][ ] km **DISTFN5**

C101 How long (hours, minutes) did it take to take him/her to the facility, receive medical care, and return home?

Tero kare ma rom mene (cawa, dakika) ki in me oo iot yat, neno darta, gamo yat, ki dok gang?

[ ][ ] hours **HRFN5**

[ ][ ] minutes **MINFN5**

C102 What mode of transport is usually used to take the child to the facility? **TRFNOW5**

Gin wot ango ma itiyo kwede me cwalo latini i ot yat?

Own bicycle	[ ] (1)	
Bicycle taxi	[ ] (2)	
Own motor bike	[ ] (3)	
Motorbike taxi	[ ] (4)	
Bus	[ ] (5)	
Special hire taxi/cab	[ ] (6)	
Borrowed bicycle	[ ] (7)	
On foot	[ ] (8)	
Other	[ ] (9)	***specify***:_____________ _______________

C103 At the child's last visit to this health facility did you have to pay fees to receive care/treatment?

I culu pi kony me yat ikare ma itero latini me agiki i ot man?

[ ] Yes = 1, No = 2 **CHFEES5**

If yes, how much did you pay in Shillings? Ka eyo, iculu ciling adi?

[ ][ ][ ][ ][ ][ ] Shs. **CHCOST5**

C104 In the last year, has the child missed any appointments for HIV care/treatment?

Imwaka ma okato ni, latini okeng ceto i ot yat i nino ma giciko me nong kony me yat?

☐ Yes = 1, No = 2 **CMISART5**

If YES, how many times? Ka eyo, tyen adii?

☐☐ Number of times **CTIMISA5**

C105 In the last year has anyone been to a health facility (with or without the child) to collect septrin for the child and been unable to?

I mwaka mukato ni ngati oceto i ot yat(ki latin onyo labong latin) ka gamo septrin , ento pe ogamo?

☐ Yes = 1, No = 2 **CUNPCOT5**

If YES (unable to pick up drugs), what is the main reason you've been unable to collect septrin?
Ka eyo, (pe ogamo yat), pi tyen lok ango ma oweko en pe ogamo yat septrin

CREASCOTP5

Drugs out of stock ☐ (1)

ART clinic was closed ☐ (2)

Referred to another facility to change treatment ☐ (3)

No health workers at facility ☐ (4)

Other ☐ (5) ***specify***:_____________

C106 In the last year has anyone been to a health facility (with or without the child) to collect ARVs for the child and been unable to?

I mwaka mukato ni ngati oceto i ot yat(ki latin onyo labong latin) ka gamo lagin , ento pe ogamo?

☐ Yes = 1, No = 2 **CUNPART5**

If YES (unable to pick up drugs), what is the main reason you've been unable to collect ARVs?
Ka eyo (pe ogamo yat), pi tyen lok ango ma oweko en pe ogamo yat lagin

CREASARTP5

Drugs out of stock ☐ (1)

ART clinic was closed ☐ (2)

Referred to another facility to change treatment ☐ (3)

No health workers at facility ☐ (4)

Other ☐ (5) ***specify***:_____________

END OF INTERVIEW
