# Supplementary material for: Population level usage of health services, and HIV testing and care, prior to decentralization of antiretroviral therapy in Agago District in rural Northern Uganda
Source: BMC Health Serv Res. 2015 Nov 28;15:527. doi: 10.1186/s12913-015-1194-4 (PMC4662831; doi:10.1186/s12913-015-1194-4)
Supplement: Additional file 2: — Population based survey individual questionnaire. (PDF 606 kb) [file 12913_2015_1194_MOESM2_ESM.pdf]

LABLITE PROJECT

POPULATION BASED SURVEY QUESTIONNAIRE

In Lira Kato sub-county, Agago District, Uganda.

Individual questionnaire

Section A: Identification

A1 Name of interviewer ______________________________________

Nying lapeny peny

A2 Interviewer code: [|] **INT**

Alama pa lapeny peny

A3 Date of interview [| |] **DINT**

Nino dwe me peny

A4 Time interview begins: [|] **TINTBEG**

Cawa ma peny ocake kwede

A5 Village number [|] **VNO**

Nama me caro

A6 Household number [| |] **HNO**

Nama me ot

A7 Name of Participant ______________________________________ **NAMEC**

Nying lagam peny

A8 Participant's identity number [| | | | | | |] **IDNO**

Lanyut pa lagam peny

Section B: Socio-demographic characteristics of the participant

NGEC IKOM LA GAM PENY

B1 Estimated date of birth day [|] month [|] year [| | |] **DOB**

Nino me nywal

B2	Sex<br>Nywal li	☐	1 = male, 2= female	**SEX**
B3	Are you in full-time education?<br>I tye ka kwan kom bedi?	☐	Yes = 1, No = 2	**FTED**

B4 What is your highest level of education? **EDUCL**

Kwan ni ma tut ogik iyi adi?

None	☐ (1)
Pre-primary	☐ (2)
Some primary	☐ (3)
Completed primary	☐ (4)
Some secondary	☐ (5)
Completed secondary	☐ (6)
Higher education	☐ (7)
Vocational	☐ (8)
Don’t know	☐ (9)

B5 What is your source of livelihood? **LIVELHD**

Ngo ma in itimo me kwo?

Livestock farmer	☐ (1)
Craftsperson	☐ (2)
Mechanic	☐ (3)
Brick maker	☐ (4)

Subsistence crop/ vegetable cultivator ☐ (5)

Cash crop grower ☐ (6)

Civil servant ☐ (7)

Alcohol brewing ☐ (8)

Tailor ☐ (9)

Miller ☐ (10)

Petty / retail business ☐ (11)

Boda boda cyclist ☐ (12)

Other ☐ (13) ***specify***:_____________ _______________

B6 Do you earn a monetary income from this livelihood?

Inwongo cente mo ikom tic me kwo ni?

☐ Yes = 1, No = 2 **MINCOME**

B7 What is your current partnership? **MSTATUS**

Kunyoni kwo ni ma calo dako onyo laco tye ni ning?

Married ☐ (1)

Living with partner as if married ☐ (2)

Never married ☐ (3)

Widow/widower ☐ (4)

Separated/divorced ☐ (5)

B8 If married or living with partner as if married, is your spouse/partner living with you now or staying elsewhere?

Ka inyome onyo itye ka kwo calo jo ma onyome, dako ni onyo cwari tye ka bedo kwedi kombedi onyo tye ka bedo kamo kene?

☐ Yes living with you = 1, No, living elsewhere = 2 **STAYPART**

B9 For men who are married or living with a partner as if married: how many wives/partners do you have who live with you?

Pi coo ma gu nyome onyo tye ka bedo ki lawote calo joo ma: itye ki mon adi ma gitye ka bedo kwedi?

☐☐ Number **NUMPART**

Section C: Health care (YOT KOME).

C1 When you have fever, where do you normally go for medical care? **FEVERFAC**

Ka itye ki lyeto pol kare i ceto kwene ka nongo yat?

(select one)

Kalongo mission hospital ☐ (1)

Private local clinic ☐ (2)

Patongo HC IV ☐ (3)

Traditional healers ☐ (4)

Lira Kato HC III ☐ (5)

Other hospital/health centre ☐ (6) ***specify***:_____________ _______________

Herbalists ☐ (7)

Other ☐ (8) ***specify***:_____________ _______________

C2 Does seeking this medical care in C1 require you to pay any fees?

Nongo kony me yat magi mite ni i cul pire?

☐ Yes = 1, No = 2 **HCFEES**

If yes, how much did you pay at your last visit? ☐☐☐☐☐☐ Shs **HCSHS**

Ka eyo, cente adi ma i culu i lim mi me agiki?

C3 What distance in kilometres do you usually have to travel to the facility?

Iwoto boo piny ma rom mene me oo i ot yat en nuni iyi km?

☐☐☐Km **DISTHC**

C4 How long (hours, minutes) does it take you to get to the facility, receive medical care, and return to your home?

Cwali kare ma rom mene(cawa/dekika) me oo i ot yat nongo kony me yat ki dwogo gang?

☐☐hours **HRSHC**

☐☐ minutes **MINSHC**

C5 What mode of transport do you usually use to get to the facility? **TRHC**

Kit gin wot mene ma pol kare itiyo kwede me oo i ot yat?

(select one)

Own bicycle	☐ (1)	
Bicycle taxi	☐ (2)	
Own motor bike	☐ (3)	
Motorbike taxi	☐ (4)	
Bus	☐ (5)	
Special hire taxi/cab	☐ (6)	
Borrowed bicycle	☐ (7)	
On foot	☐ (8)	
Other	☐ (9)	***specify***:_____________ _______________

C6 Do you suffer from any of the following conditions; High Blood Pressure, Diabetes or sugar diseases, Arthritis, Asthma, Heart attack, or any other heart disease?

Itye ki peko me two egi ni; Pressure, two cukari, arem me kom, Asthma,two adunu, /kit two cwiny mo?

☐ 1=yes,2=no,3=don't know **CHRONIC**

If yes where do you usually access care? **HCACC**

Ka eyo pol kare i nongo kony me yat ki kwene?

(select one)

Kalongo mission hospital	☐ (1)
Private local clinic	☐ (2)
Patongo HC IV	☐ (3)
Traditional healers	☐ (4)
Lira Kato HC III	☐ (5)
Other hospital/health centre	☐ (6) ***specify***:____________ ______________
Herbalists	☐ (7)
Other	☐ (8) ***specify***:____________ ______________

C7 What was the last serious sickness you had which required medical care in the last 12 months? **ILL**

Two mene ma oyeli me agiki marac ma oweko inongo kony me yat?

(select one)

Malaria	☐(1)
Diabetes M/sugar	☐(2)
High blood pressure/hypertension	☐(3)
Heart disease	☐(4)
Tuberculosis	☐(5)
None	☐(6)
Other	☐ (7) ***specify***:____________ ______________

If C7 is NONE then skip to C9

C8 Where did you go for medical care for that illness? **CAREILL**

Ka kwene ma iceto ka nongo yat pi two enoni?

(select one)

Kalongo mission hospital	☐ (1)
Private local clinic	☐ (2)
Patongo HC IV	☐ (3)
Traditional healers	☐ (4)
Lira Kato HC III	☐ (5)
Other hospital/health centre	☐ (6) ***specify***:____________ ______________
Herbalists	☐ (7)
Other	☐ (8) ***specify***:____________ ______________

C9 Has illness kept you away from your normal work for at least a month in the last one year?

Two ogengi tic ci majwir ma romo pi dwe acel ikin maka acel ma okato ni?

☐ Yes = 1, No = 2 **AWAYILL**

C10 How long have you spent away from work in the last month as a result of ill health?

I bedo pi kare ma rom mene labongo tic i dwe ma okatoni ma lube ki two ni?

☐☐ number of days **DAYSILL**

C11 Have you had to sell any assets to pay for health care for you or any member of your family in the last 3 months?

Tika dong i cato jami ni ma pire tek me culu pi two ni onyo pa nget mo ma i gangi ikine ka dwe adek ma okato ni?

☐ Yes = 1, No = 2 **SELLTOILL**

C12 Have you had to borrow money from the community to pay for health care for you or your family in the last 3 months?

Tika dong i deno cente ki ikin gang me culu pi two ni onyo pa ngat mo ma igangi ikine ka dwe adek ma okato ni?

☐ Yes = 1, No = 2 **OWETOILL**

If yes, have you since repaid this money? Ka eyo, tika dong i culu?

☐ Yes = 1, No = 2 **PAIDTOILL**

C13 How many times have you visited each of the following health facilities for health care in the last 3 months? Tyen adi ma dong i limo odi yadi egini me nongo kony me yat i kine ka dwe adek ma okato ni?

(Answer for each facility)

Kalongo mission hospital	☐☐	number of times/0=none	**KAL**
Private local clinic	☐☐	number of times/0=none	**PRIVATE**
Patongo HC IV	☐☐	number of times/0=none	**PAT**
Traditional healers	☐☐	number of times/0=none	**TRAD**
Lira Kato HC III	☐☐	number of times/0=none	**LIRAK**
Other hospital/health centre	☐☐	number of times/0=none	**PUBLIC**
Herbalists	☐☐	number of times/0=none	**HERB**
Other	☐☐	number of times/0=none	**OTHTI**

Section D: HIV Knowledge and Prevention

(Ngec ikom kwidi me two jonyo ki yo me gengo)

D1 Have you heard of HIV or AIDS? ☐ Yes = 1, No = 2. **KNOWHIV**

Tika dong i winyo pi kwidi me two jonyo onyo two jonyo?

D2 A lot of things have been said about ways of transmitting HIV/AIDS; through which of the following ways do you think HIV/AIDS can be transmitted? Jami ma pol dong ki Waco ikom kit ma kwidi me two jonyo kobo kwede; kit yoo mene ma itamo ni kwidi me two jonyo twero kobo kwede?

Read the list to respondent and code 1=Yes, 2= No, 3=don't know/not sure

Having sex with someone who looks healthy	☐	**REASAIDS1**
Mosquito/or other insect bites	☐	**REASAIDS2**
Bewitchment/curses or other supernatural means	☐	**REASAIDS3**
Sharing food with a person who has the HIV	☐	**REASAIDS4**
Having sex with someone without a condom	☐	**REASAIDS5**

D3 Do you practise any HIV prevention methods? In ilubu kit yoo mo me gwoke ki two jonyo?

☐ Yes = 1, No = 2, 9 = N/A **PREVHIV**

D4 Which of these HIV prevention practices have you used? Kit yoo mene me gwoke ki two jonyo ma i tiyo kwede?

Read the list to respondent and code each

Abstinence	☐	1=yes,2=no,3=don't know	**ABST**
Faithfulness	☐	1=yes,2=no,3=don't know	**FAITH**
(Limiting sexual intercourse to one uninfected partner)			
Condom use	☐	1=yes,2=no,3=don't know	**COND**
Circumcision	☐	1=yes,2=no,3=don't know	**CIRC**
Other specify	☐	1=yes,2=no,3=don't know	**OTHPREV**

specify:____________ ______________

If not used any method above reason for not using? Ka pe otiyo ki yoo moo keken ikin ma malo ni nong pingo ne?

Read the list to respondent and code each

Religious beliefs	☐	1=yes,2=no,3=don't know	**REASNUSE1**
My partner does not like condoms	☐	1=yes,2=no,3=don't know	**REASNUSE2**
Cannot afford condoms	☐	1=yes,2=no,3=don't know	**REASNUSE3**
I cannot stay with one partner only	☐	1=yes,2=no,3=don't know	**REASNUSE4**
I cannot abstain	☐	1=yes,2=no,3=don't know	**REASNUSE5**
Other specify	☐	1=yes,2=no,3=don't know	**REASNUSE6**

specify:_____________ _______________

D5 Can the HIV be transmitted from the following?

Tika kwidi me two jonyo twero kobo iyo magi?

Mother to child during pregnancy	☐	1=yes,2=no,3=don't know	**MTCT1**
Mother to child during delivery	☐	1=yes,2=no,3=don't know	**MTCT2**
Mother to child during breastfeeding	☐	1=yes,2=no,3=don't know	**MTCT3**

If any yes on MTCT, how can one reduce the risk of transmission from mother to child?

Ka mo tye eyo ki i MTCT, Ngat mo twero dwoko kero me kobo pa two jonyo ninig bot latin?

☐ 1=By taking special drugs during pregnancy **PMTCT1**

2=Don't know/not sure

D6 Can you please tell me where one can get information on prevention of HIV in this parish?

Iromo wac ca kama ngat mo romo nwongo ngec ikom kit me gwoke ki kwidi two jonyo ite mukumu ni?

Read the list and code each 1=Yes, 2= No, 3=don't know/not sure

Govt/mission health unit	☐	**INFOR1**
Private clinic	☐	**INFOR2**

Family planning office	☐		**INFOR3**
Pharmacy / drug shop	☐		**INFOR4**
Retail shop	☐		**INFOR5**
Bar	☐		**INFOR6**
Schools	☐		**INFOR7**
Other	☐	Spe***cify***:_________	**INFOR8**

D7 How many partners have you had sex with without a condom in the last 3 months?

I butu ki luwoti adi labongo tic ki roc bol ikine me dwe adek angec?

☐☐number **NCOND**

Section E: Antenatal Care (Pregnancy and child birth). THIS SECTION IS FEMALES ONLY

E1 Have you ever been pregnant? ☐ Yes = 1, No = 2. **PREG**

In dong iyac?

If no, go to section F

E2 How many times have you ever become pregnant, including abortions and miscarriages/stillbirths?

Dong iyac tyen adi, ii oony tyen adi onyo obale onyo inywalo ma oto?

☐☐times **PREG1**

E3 How many births have you had (include still and live births but not miscarriages or abortions)?

Dong inywalo odok otino adu? (Jo akwo kede en otoo) Ikare me nywal.

☐☐ number BIRTH1

IF NONE SKIP TO SECTION F

E4 What was the outcome of the last birth? Adwogi me yacu no obedo ninig?

☐ Still birth = 1, Live birth = 2 **PREGOUTC**

If live birth has your child had measles vaccinations in the first year of his/her life?
Ka latin makwo dong i gwero latin ni pi two anyoo imwaka ne me acel?

☐ Yes = 1, No = 2, 3= Don't know/cannot remember, 4=child<1year. **MSVACC**

If yes, where did you access this service? **MEASACC**
Ka eyo, inongo kony ma ki kwene?

Kalongo mission hospital	☐ (1)
Private local clinic	☐ (2)
Patongo HC IV	☐ (3)
Traditional healers	☐ (4)
Lira Kato HC III	☐ (5)
Other hospital/health centre	☐ (6) ***specify***:_____________ _______________
Herbalists	☐ (7)
Other	☐ (8) ***specify***:_____________ _______________

E5 In what month and year did you have your last birth?
Nywal li me agiki obedo i mwaka mene ki dwe mene?

☐☐ month **MTHPREG**

☐☐☐☐ year **YRPREG**

WE ARE NOW GOING TO ASK A SERIES OF QUESTIONS RELATING TO THIS LAST BIRTH.

KOM BEDI DONG WA BI PENYI LAPENY MA DWONG MA LUBE KI NYWALI ME AGIKI.

If before 2008 skip rest of section go to F

E6 Did you attend antenatal clinic during the pregnancy leading to your last birth?

Ipime iyaco ni ma inywal kede me agiki ni?

☐ Yes = 1, No = 2, 9 = Don't know. **ANC**

IF NO GET REASON AND SKIP TO SECTION F

Ka mapud pi 2008 kal lapeny mogo ni weng wa i F

If no, What was the main reason you did not attend antenatal clinic? **REASANC**

Ka ku, pi ngo pe ipime?

Clinic too far ☐ (1)

Had no money ☐ (2)

Had no time ☐ (3)

Not aware had to attend ☐ (4)

Did not want to attend ☐ (5)

Other ☐ (6) ***specify***:_____________ _______________

E7 How many months pregnant were you when you first attended ANC during the pregnancy leading to your last birth?

I cako pime ki dwe adi iyaco ni ki nywal ma agiki ni?

☐ months **MTHANC1**

E8 How many times did you attend antenatal clinic during the pregnancy leading to your last birth?

Ipime tyen adi iyaco ni ki nywal ma agiki ni?

☐☐ times **NUMANCV1**

E9 Where did you usually attend antenatal clinic? **ANCFAC**

Pol kare ipime kwene?

Kalongo mission hospital ☐ (1)

Private local clinic ☐ (2)

Patongo HC IV ☐ (3)

Lira Kato HC III ☐ (4)

Other hospital/health centre ☐ (5) ***specify***:____________ ______________

E10 Did you pay fees to attend antenatal clinic? I culu cul mo me pime?

☐ Yes = 1, No = 2, 3 = Don't know. **ANCFEES1**

If yes, how much did you pay for each visit to the antenatal clinic? Ka eyo, i culu ciling adi pi pime acel acel?

☐☐☐☐☐☐ Shs. **ANCSHS**

E10 Were you tested for the HIV virus as part of your antenatal care?

Tika ki pimi pi two jonyo ikare me pime?

☐ Yes = 1, No = 2, 8= Don't know/cannot remember. **ANCTEST1**

If Yes, go to E11 (Ka eyo ceti i E11)

If no, were you offered a test for the HIV virus as part of your antenatal care? Ka ku, tika ki pimi pi kwidi me two jonyo calo dul me pime ni?

☐ Yes = 1, No = 2, 8= Don't know/cannot remember. **ANCTEST2**

If yes to offered a test **ie ANTEST2**,(GET REASON WHY AND SKIP TO SECTION F) why did you not take the test? **REASTEST2**

Ka eyo ma onongo kony me pime iye ANTEST2, (Nong pingone ci ikal i F) Pi ngo pe ipime?

(select one)

I don't want to know my HIV results ☐ (1)

I fear being tested for HIV ☐ (2)

I am not at risk ☐ (3)

Counseling offices are too far ☐ (4)

There is no need ☐ (5)

I'm not interested ☐ (6)

I've never thought about it ☐ (7)

Other ☐ (8) ***specify***:____________

E11 If you had an HIV test done as part of your antenatal care, where was the test done?
Ka ki pimi pi kwidi me tyo jonyo ikare me pime ni otime ki kwene?

ANCTFAC

(select one)

Kalongo mission hospital ☐ (1)

Private local clinic ☐ (2)

Patongo HC IV ☐ (3)

Lira Kato HC III ☐ (4)

Other hospital/health centre ☐ (5) ***specify***:____________ ______________

E12 When was the test done? Ki pimi awene?

☐☐ month **MTHATEST**

☐☐☐☐ year **YRANTEST**

Check If done at ANC (E9 and E11 same) clinic ignore question below on distance SKIP TO E16

Ngii ka kitimo i ANC (E7 ki E11 rom) kilinic wek lapeny ma pinyi ci ikale i E16

E13 What distance in kilometres did you have to travel to the facility that did your HIV test? Iwoto boo piny ma rom mene iyi (km) me oo kama inong kony me pime pi kwidi two jonyo?

☐☐☐ km **DISTANC**

E14 How long (hours, minutes) did it take you to get to the facility, to have an HIV test medical care, and return to your home? Ocwali kare marom mene (cawa, dekika) me oo i ot yat, pime pi kwidi me two jonyo ki dwogo gang?

☐☐ hours **HRSANC**

☐☐ minutes **MINSHC1**

E15 What mode of transport did you use to get to the facility? **TRANC**

Itiyo ki gin wot ango?

(select one)

Own bicycle	☐ (1)	
Bicycle taxi	☐ (2)	
Own motor bike	☐ (3)	
Motorbike taxi	☐ (4)	
Bus	☐ (5)	
Special hire taxi/cab	☐ (6)	
Borrowed bicycle	☐ (7)	
On foot	☐ (8)	
Other	☐ (9)	***specify***:____________ ______________

E16 Did you get the result of the test? Inongo adwoki me pime ni?

☐ Yes = 1, No = 2 **GETRES**

If No, (GET REASON WHY AND SKIP TO SECTION F) why did you not get the result? Ka ku, (nong tyen lok ke ci kale i SECTION F) pi ngo pe igamo adwoki me pime ni?

REASNRES

I didn't want to know my HIV results ☐ (1)

I feared being tested for HIV ☐ (2)

I was not at risk ☐ (3)

Counseling offices were too far ☐ (4)

There was no need ☐ (5)

I was not interested ☐ (6)

I've never thought about it ☐ (7)

Other ☐ (8) ***specify***:____________

If yes, what was the result of the test? ka eyo, adwoki ne obedo ninig?

☐ Positive = 1, Negative = 2, 3=Refused to answer **PREGRES**

If positive, following this test were you referred to a health facility for HIV care for your own health? Ka tye, ma lube ki pime eni tika ki cwali i ot yat pi nongo kony pi kwidi me two jonyo pi yot komi?

☐ Yes = 1, No = 2 **ANCREFER**

If negative, have you had another test since this one?

Ka ku, tika dak inongo pime mukene inge eni?

☐ Yes = 1, No = 2 **OTHTEST**

IF NO STOP, (ka ku gik kany,)

IF YES SKIP TO SECTION F, QUESTION 2 (ka eyo cet i section f, lapeny me 2)

PLEASE ONLY ASK IF TESTED POSITIVE(E16) AND LIVE BIRTH (E9)

PENY KEKEN KA KIPIMO KI NONGO TYE KI KWIDI ME TWO JONYO (E16) DOK ONYWALO LATIN MA KWO (E9)

E17 If positive (including women who had previously tested positive): During the pregnancy or during labour and delivery, were you offered anti-retroviral medicines (ARVs) to reduce the risk of

passing on the AIDS virus to your unborn baby?
Ka tye (wa mon ma con ma tye ki two jonyo): ikare me yacu, arem ki nywali , ki mini yat lagin me dwoko kero me kobo pa kwidi two jonyo ikom latin ma iyic?

☐ Yes = 1, No = 2 **OFFMTCT**

If yes, did you take the drugs? Ka eyo, i mwunyu yat te?

☐ Yes = 1, No = 2 **ARTMTCT**

E18 Was your baby also given treatment to prevent HIV infection? Latin ni bene ki mine yat me gengo kwidi me two jonyo?

☐ Yes = 1, No = 2 **ARTTOBABY**

FOR WOMEN WHO TESTED POSITIVE SKIP TO SECTION G
Pi mon ma dong tye ki kwidi me two jonyo cet i G

Section F: HIV TESTING AND UPTAKE OF SERVICES.

Can skip all this section for women who've already reported testing positive under ANC questions

F1 Have you ever taken an HIV test? In dong tika i pime pi kwidi me two jonyo?

☐ Yes = 1, No = 2, 3 = Refused to answer **EVERTEST**

If not, collect reason then stop interview **REASNT1**
Ka ku, nong tyen lok ke ci juk lapeny
(select one)

I don't want to know my HIV results ☐ (1)

I fear being tested for HIV ☐ (2)

I am not at risk ☐ (3)

Counseling offices are too far ☐ (4)

There is no need ☐ (5)

I'm not interested ☐ (6)

I've never thought about it ☐ (7)

Other ☐ (8) ***specify***:____________

F2 When was the last time you were tested? Ipime me agiki awene?

☐☐ month **MTHTES**

☐☐☐☐ year **YRTEST**

Month and year if within the last 2 years OR year if more than 2 years ago

F3 Where did you have your most recent test? **RECTEST**

Kwene ma ipime iye me agiki?

(select one)

Kalongo mission hospital ☐ (1)

Private local clinic ☐ (2)

Patongo HC IV ☐ (3)

Traditional healers ☐ (4)

Lira Kato HC III ☐ (5)

Other hospital/health centre ☐ (6) ***specify***:____________ ______________

Herbalists ☐ (7)

Other ☐ (8) ***specify***:____________ ______________

F4 Did you have to pay for the test? Iculu cul mo pi pime?

☐ Yes = 1, No = 2 **PAIDTEST**

If yes, how much did you pay for the test in Shillings? Ka eyo, i culu cente adi?

☐☐☐☐☐☐ Shs. **TESTCOST**

F5 What distance in kilometres did you have to travel to the facility?
I woto boo piny ma rom mene i (km) me oo i ot yat?

☐☐☐ Km **DISTEST**

F6 How long (hours, minutes) did it take you to get to the facility for an HIV test, have an HIV test, and return to your home? Ocwali kare marom mene (cawa, dekika) me oo i ot yat, pime pi kwidi me two jonyo ki dwogo gang?

☐☐ hours **HRSTEST**

☐☐ minutes **MINSTEST**

F7 What mode of transport did you use to get to the facility? **TRTEST**

Itiyo ki gin wot ango me oo i ot yat?

(select one)

Own bicycle	☐	(1)
Bicycle taxi	☐	(2)
Own motor bike	☐	(3)
Motorbike taxi	☐	(4)
Bus	☐	(5)
Special hire taxi/cab	☐	(6)
Borrowed bicycle	☐	(7)
On foot	☐	(8)
Other	☐	(8) ***specify***:_____________ _______________

F8 Did you receive your HIV test results? Inongo adwoki me pime ni?

☐ Yes = 1, No = 2, 3= Refused to answer **RECTRES**

If no, why not? IF NO GET REASONS AND STOP HERE **REASNTR1**

Ka ku pi ngo, ka ku nong tyen lok ke ci i gik kany

(select one)

I changed my mind ☐ (1)

Results did not come back ☐ (2)

I already knew my HIV results ☐ (3)

Counseling offices were too far ☐ (4)

Results took too long, became irrelevant ☐ (5)

Other ☐ (6) ***specify***:____________

If yes, what was the result of the test?

☐ Positive = 1, Negative = 2, 3= Refused to answer **TESTRES**

IF NEGATIVE STOP HERE

KA PEKE KI TWO GIK KO

Section G: HIV POSITIVES.

G1 Have you ever enrolled in HIV care, for treatment with Septrin or ART?

Tika dong I coo ne pi kony me kwidi two jonyo, nwongo yat septrin onyo lagin?

☐ Yes = 1, No = 2 **ENROLHIV**

If never attended a health facility for HIV care, why not? ie IF NEVER ATTENDED GET REASON AND STOP HERE (Ka pud piya i oo i ot yat pi nwongo kony pi kwidi me two jonyo, nong pingo ne ci gik kany)

REASNENR1

<u>(select one</u>)

Transport costs ☐ (1)

Lack of drugs ☐ (2)

Stigma ☐ (3)

Copayments/user fees ☐ (4)

Never referred to a health facility ☐ (5)

I feel healthy ☐ (6)

Other ☐ (7) ***specify***:_____________ _______________

G2 When did you last attend a health facility for HIV care? Ki pimi me agiki awene?

☐☐ month **MTHHIVC**

☐☐☐☐ year **YRHIVC**

G3 Have you attended a health facility for HIV care in the last 12 months?

Tika I nwongo kony me ot yat ikom kwidi mw two jonyo ikine me dwe apararyo ni?

☐ Yes = 1, No = 2 **HIVC12M**

If no, why not? ie IF NO, GET REASONS AND STOP HERE **REASNATT1**

Ka ku, pi ngo, nwong pi ngo ne ci gik lapeny

Transport costs ☐ (1)

Lack of drugs ☐ (2)

Stigma ☐ (3)

Co-payments/user fees ☐ (4)

Other ☐ (5) ***specify***:_____________ _______________

G4 Name of facility where you currently receive care? **HFAC12**

Ot yat mene ma kunyoni i nwongo iye kony me yat?

Lira kato HC III ☐ (1)

Patongo HC IV ☐ (2)

Kalongo mission hospital ☐ (3)

Other ☐ (4) ***specify***:____________ ______________

G5 At your last visit to this health facility did you have to pay fees?

Ilim mi ma agiki i ot yat i culu cente moo?

☐ Yes = 1, No = 2 **HIVCFEES**

If yes, how much did you pay in Shillings? Ka eyo,i culu cente adi?

☐☐☐☐☐☐ Shs. **HIVCCOST**

G6 What distance in kilometres do you have to travel to this health facility to receive HIV care?

I woto boo piny ma rom mene i (km) me oo i ot yat?

☐☐☐ Km **DISTHIVC**

G7 How long (hours, minutes) does it usually take you to get to this health facility, receive HIV care at the facility, and travel back to your home? Ocwali kare marom mene (cawa, dekika) me oo i ot yat, pime pi kwidi me two jonyo ki dwogo gang?

☐☐ hours **HRSHIVC**

☐☐ minutes **MINSHIVC**

G8 What mode of transport do you usually use to get to this health facility? **TRHIVC**

Itiyo ki gin wot ango me oo i ot yat?

(select one)

Own bicycle ☐ (1)

Bicycle taxi ☐ (2)

Own motor bike ☐ (3)

Motorbike taxi ☐ (4)

Bus ☐ (5)

Special hire taxi/cab ☐ (6)

Borrowed bicycle ☐ (7)

On foot ☐ (8)

Other ☐ (9) ***specify***:____________ ______________

G9 At your last visit to this health facility, did someone travel with you?

Ilim mi me agiki i ot yat i woto ki gat mo?

☐ Yes = 1, No = 2 **HIVCPART1**

If YES, who travelled with you to this health facility? **HIVCPART2**

Ka eyo, nga ma wu woto kwede i ot yat?

Spouse ☐ (1)

Child ☐ (2)

Brother ☐ (3)

Sister ☐ (4)

Father ☐ (5)

Mother ☐ (6)

Friend ☐ (7)

Neighbour ☐ (8)

Other ☐ (9) ***specify***:____________ ______________

G10 Do you know any of these drugs? GET PICTURES Ingeyo kwai yadi egi ni nyut cal gi?

SEPTRIN ☐ Yes = 1, No = 2 **COTOX**

ISONIAZIDE ☐ Yes = 1, No = 2 **INH**

(Lajin) ART OR ARVS? ☐ Yes = 1, No = 2 **ART**

G11 Which of these drugs are you taking/have you taken?

Ikin yadi ni mene ma itye ka mwunyo/onyo ma imwunyo?

SEPTRIN ☐ Never taken = 1, **ONCOTOX**

Taken in the past but not currently taking = 2,

Currently taking =3

ISONIAZIDE ☐ Never taken = 1, **ONINH**

Taken in the past but not currently taking = 2,

Currently taking =3

ARVS ☐ Never taken = 1, **ONART**

Taken in the past but not currently taking = 2,

Currently taking =3

If currently taking Septrin, when did you start Septrin?

Ka itye ka mwunyo Septrin, awene ma icako mwunyo Septrin?

month ☐☐ **MTHCOT**

year ☐☐☐☐ **YRCOT**

If currently taking Isoniazide, when did you start Isoniazide?

Ka itye ka mwunyo Isoniazide, awene ma icako mwunyo Isoniazide?

month ☐☐ **MTHINH**

year ☐☐☐☐ **YRINH**

If currently taking ARV's, when did you start ARVs?

Ka itye ka mwunyo lajin, awene ma icako mwuno lajin?

month ☐☐ **MTHART**

year ☐☐☐☐ **YRART**

G12 How often do you (actually) go to pick up your drugs?

Pol kare i ceto ka gamo yat ti inge kare marom mene?

months ☐☐ SEPTRIN **MTHPCOT**

months [ ][ ] ISONIAZIDE **MTHPINH**

months [ ][ ] ARV's **MTHPART**

G13 Does anyone else pick up your drugs for you? Tika ngat ti mo gamo yat ti?

[ ] Yes = 1, No = 2 **OTHPICK**

If Yes, number of times someone has picked up drugs for you in the last 12 months

Ka eyo, tyen adi ma ogami yat ti ikin dwe apararyo ma okato angec?

[ ][ ] Number of times **TIMESP**

G14 For how many days/weeks=MONTH supply of drugs are you given at each clinic visit?

Pi nino ma rom mene/cabit onyo dwe ma ki mi ni yat ti ki i ot yat?

months [ ][ ] SEPTRIN **MTHCOTSS**

months [ ][ ] ISONIAZIDE **MTHINHSS**

months [ ][ ] ARV's **MTHARTSS**

IF NOT ON ART SKIP TO G16 (KA PEKE IYI LAJIN KAL I G16)

G15 If on ART, in the past year have you missed going to the clinic to pick up ARVs due to distance or cost? Ka tye ka mwunyo lajin, i mwaka ma okato ni tika ikeng ceto i ot yat ka gamo yat lajin ni pi boo piny onyo pi wele matek?

[ ] Yes = 1, No = 2 **MISSART12**

If YES, how many times? Ka eyo, tyen adi?

[ ][ ] Number of times **TIMESMIS**

G16 In the last year have you gone to the facility where you currently receive care and been unable to pick up drugs? imwaka acel ma okato ni nino mo tye ma i ceto ka nwongo kony me yat ci pe inongo?

[ ] Yes = 1, No = 2 **NTPICK**

If yes, how many times? Ka eyo, tyen adi?

Number of times ☐☐ SEPTRIN **UNPSEP**

Number of times ☐☐ ISONIAZIDE **UNPISO**

Number of times ☐☐ ARV's **UNSART**

If unable to pick up drugs, what is the main reason you've been unable to collect drugs?

Ka pe onongo yat, tyen lok ango kikome ma ogengi nongo yat?

REASUNP1

Drugs out of stock	☐ (1)	
ART clinic was closed	☐ (2)	
Referred to another facility to change treatment	☐ (3)	
No health workers at facility	☐ (4)	
Other	☐ (5)	***specify***:____________

G17 Has your blood been taken in the last 12 months as part of your HIV care?

Tika remoni kipimo i kine dwe apararyo macalo yoo me gwoke ni i kom two jonyo?

☐ Yes = 1, No = 2 **BLDTAKE**

If yes, do you know why your blood was taken? Ka eyo, inyeyo piongo kikwanyo remoni?

REASBLD1

CD4 COUNT	☐ (1)
Referred to another facility to change treatment	☐ (2)
Malaria	☐ (3)
Don't know	☐ (4)

G18 Was your blood taken at the facility where you currently receive care the last time your blood was taken? I agiki ma kikwanyo remoni ni tika remoni kicwalo i ot yat kama kunyoni itye kanongo kony me yat iiye ni?

☐ Yes = 1, No = 2 **BLDATFAC**

If no, where was your blood taken the last time you had a blood test? **BLDHFAC**

Ka kukwene makikwanyo remoni i me agiki maipime,?

Kalongo mission hospital	☐ (1)	
Lira Kato HC III	☐ (2)	
Patongo HC IV	☐ (3)	
Other	☐ (4)	***specify***:____________ ______________

G19 At your last visit to this health facility did you have to pay fees?
Ilim mi ma agiki i ot yat t ii culu cente moo?

☐ Yes = 1, No = 2 **BLDFEES**

If yes, how much did you pay in Shillings? Ka eyo,i culu ciling adi

☐☐☐☐☐☐ Shs. **OTHCOST**

G20-G22 only apply if not at current facility ie G18 IS No. (G20-G22 rwate keken ka peke i ot yat ma kom bedi en aye G18 ku)

G20-G22 all refer to last blood test (G20-G22 weng loko ikom pimo remo me agiki)

G20 What distance in kilometres do you have to travel to this health facility to receive a blood test?
I woto boo piny ma rom mene i (km) me oo i ot yat?

☐☐☐ Km **DISTBLD**

G21 How long (hours, minutes) did it take you to get to this health facilityhave a blood test, and travel back to your home? Ocwali kare marom mene (cawa, dekika) me oo i ot yat, pime pi kwidi me two jonyo ki dwogo gang?

☐☐ hours **HRSBLD**

☐☐ minutes **MINSBLD**

G22 What mode of transport did you use to get to this health facility? **TRBLD**

Itiyo ki gin wot ango me oo i ot yat?

Own bicycle ☐ (1)

Bicycle taxi ☐ (2)

Own motor bike ☐ (3)

Motorbike taxi ☐ (4)

Bus ☐ (5)

Special hire taxi/cab ☐ (6)

Borrowed bicycle ☐ (7)

On foot ☐ (8)

Other ☐ (9) ***specify***:____________ ______________

G23 Did you go to this facility (that you are currently receiving care) the first time you enrolled into HIV care? Tika i ceto i ot yat ma kunyoni i gamo iye yat ti iacaki ma icone pi kony me kwidi two jonyo?

☐ Yes = 1, No = 2 **ENROLFAC**

If YES, SKIP to G24, (Ka eyo, cet i G24)

If NO, collect the reason why moved **REASMOVEC1**

Ka ku, nong pi ngo okobo

Transferred ☐ (1)

Lack of drugs ☐ (2)

Other ☐ (3) ***specify***:____________ ______________

If reason is transfer, collect reason for transfer **REASTRANS**

Ka okobo, pi ngo okobo

Moved nearer to home ☐ (1)

Lack of drugs ☐ (2)

Other ☐ (3) ***specify***:____________ ______________

What is the name of facility you attended prior to this one? **FACNAME**

Ot yat mene ma i oo iye ma pud piya i oo kany?

Kalongo mission hospital ☐ (1)

Lira Kato HC III ☐ (2)

Patongo HC IV ☐ (3)

Other ☐ (4) ***specify***:____________ ______________

G24 In the last year (after enrolling at this facility) have you been to any other health facilities for HIV care NOT TRANSFERS? I mwaka ma okato ni tika i oo i ot yat mukene pi kony me kwidi two jonyo ma pe ki kobi akoba?

☐ Yes = 1, No = 2 **COENR**

If YES, give reasons why, **REASCOENR1**

Ka eyo, pi ngo

Closer to home ☐ (1)

Stigma ☐ (2)

Lack of drugs ☐ (3)

Referred by health workers from primary facility ☐ (4)

Other ☐ (5) ***specify***:____________________

END OF INTERVIEW
